# Colorectal Cancer Care and Patient’s Perceptions Before and During COVID-19: Implications for Subsequent SARS-CoV-2 Infection Waves

**DOI:** 10.1093/jncics/pkab047

**Published:** 2021-05-13

**Authors:** Jeroen W G Derksen, Anne M May, Lonneke V van de Poll-Franse, Belle H de Rooij, Dorothee A Hafkenscheid, Helena M Verkooijen, Miriam Koopman, Geraldine R Vink, PLCRC study group

**Affiliations:** 1 Julius Center for Health Sciences and Primary Care, University Medical Center Utrecht, Utrecht University, Utrecht, The Netherlands; 2 Department of Medical Oncology, University Medical Center Utrecht, Utrecht University, Utrecht, The Netherlands; 3 Department of Research and Development, Netherlands Comprehensive Cancer Organisation (IKNL), Utrecht, the Netherlands; 4 Division of Psychosocial Research & Epidemiology, The Netherlands Cancer Institute, Amsterdam, the Netherlands; 5 Department of Medical and Clinical Psychology, Center of Research on Psychological and Somatic, Disorders (CoRPS), Tilburg University, Tilburg, the Netherlands; 6 Imaging Division, University Medical Center Utrecht, Utrecht University, Utrecht, The Netherlands

**Keywords:** COVID-19, Colorectal cancer, Quality of life, Cancer care

## Abstract

**Background:**

Changes in colorectal cancer (CRC) care planning due to the coronavirus disease 2019 (COVID-19) pandemic and associated health-related quality of life (HRQoL) and well-being of patients with CRC are unknown. We report changes in CRC care and patient-reported outcomes (PROs) including HRQoL, distress, and loneliness during the first wave of SARS-CoV-2.

**Methods:**

In April 2020, 4,984 patients included in the nationwide Prospective Dutch Colorectal Cancer cohort were invited to complete a COVID-19-specific questionnaire, together with the validated EORTC QLQ-C30, De Jong Gierveld, and HADS. Clinical data were obtained from the Netherlands Cancer Registry. Scores were compared with the year prior to COVID-19, and with an age- and sex-matched control population during COVID-19.

**Results:**

In total, 3,247 (65.1%) patients responded between April and June 2020. Seventeen percent of patients had cancelled/postponed/changed hospital visits into a telephone- or video consult while 5.3% had adjusted/postponed/cancelled treatment. Compared to controls, patients reported worse HRQoL, but comparable distress and less social loneliness (patients = 21.2%; controls = 32.9%). Compared to pre-COVID-19, clinically meaningful deterioration of HRQoL was more prevalent in patients with changes in cancer care planning than in patients without changes. Prior to undergoing or currently undergoing treatment, and infection worries were associated with lower HRQoL.

**Conclusions:**

CRC patients reported equal anxiety and depression, but worse HRQoL than the control population. Changes in care planning were associated with deterioration of HRQoL and increased anxiety. In case of one or more risk factors, healthcare specialists should discuss (mental) health status and possible support during future SARS-CoV-2 infection waves or comparable pandemics.

**Abbreviations**

| | |
|---|---|
| BMI | Body mass index |
| COVID-19 | Coronavirus disease 2019 |
| CRC | Colorectal cancer |
| EORTC | European Organisation for Research and Treatment of Cancer |
| HADS | Hospital Anxiety and Depression Scale |
| HRQoL | Health-related quality of life |
| ICD-10 | International Classification of Diseases, tenth edition |
| MREC | Medical Research Ethics Committee |
| NCR | Netherlands Cancer Registry |
| NFK | Dutch federation of cancer patient organisations |
| PLCRC | Prospective Dutch colorectal cancer cohort |
| PRO | Patient-reported outcomes |
| PROFILES | The Patient Reported Outcomes Following Initial treatment and Long term Evaluation of Survivorship registry |
| SARS-CoV-2 | Severe Acute Respiratory Syndrome coronavirus 2 |
| TCI | Thresholds for clinical importance |
| TC | Telephone consultation |
| TNM | Tumor, Node, Metastases classification system |
| VC | Video consultation |

### Keywords

COVID-19, Colorectal cancer, Quality of life, Cancer care

In March 2020, shortly after the outbreak of the COVID-19 pandemic, hospitals were suddenly required to reorganize in order to optimize clinical care for patients with COVID-19 and lower the risk of infection for all others involved. Consequently, ensuring the timely and optimal non-COVID-19 care has become increasingly challenging. Moreover, due to the absence of herd immunity and incomplete administration of all inhabitants with an effective COVID-19 vaccine, most countries will face multiple "waves" of infections. Current projections estimate that the COVID-19 pandemic could last years, possibly leading to prolonged or intermittent measures ranging from social distancing to (partial) lockdowns, ultimately to maintain intensive care capacity [1]. Therefore, it is important to ensure that patients continue to receive essential care while minimizing their risk of SARS-CoV-2 infection, and to evaluate how initial clinical reorganizations were perceived by patients in terms of health-related quality of life (HRQoL) and mental well-being.

Here we aim to 1) evaluate changes in CRC treatment and follow-up care during the first wave of SARS-CoV-2 infections in The Netherlands, 2) compare HRQoL and well-being of CRC patients with an age- and sex-matched non-cancer control population, 3) study whether CRC patients experience clinically meaningful changes in HRQoL and mental well-being during the COVID-19 outbreak, as compared to the year before COVID-19, and 4) investigate which sociodemographic and clinical factors are related to changes in PROs.

### Methods

#### Design and setting

The current study is an observational study embedded in the ongoing nationwide Prospective Dutch Colorectal Cancer (PLCRC) cohort; an initiative coordinated by the Dutch Colorectal Cancer Group. PLCRC consists of patients diagnosed with a malignancy of the colon and/or rectum (ICD-10: C18-20) in the Netherlands. The PLCRC study protocol, described in more detail elsewhere [2], has been approved by the Medical Research Ethics Committee (MREC) Utrecht, the Netherlands. At PLCRC entry, participants provide informed consent and can opt to receive longitudinal questionnaires, which allows for a swift implementation of questionnaire-based observational studies. Clinical data was obtained through linkage with the Netherlands Cancer Registry (NCR).

#### Study population

In total, 4,984 PLCRC participants who provided informed consent for receiving questionnaires were invited to participate in the current study and received an additional COVID-19-specific questionnaire on oncologic practice patterns, cancer care, HRQoL and well-being during the COVID-19 outbreak. During the first national lockdown from March 23 until June 1, 2020, the COVID-19-specific questionnaire was sent out on April 17, 2020 and was received until June 1, 2020. Patients were included in the longitudinal analysis when a previous questionnaire of PLCRC was completed in the year before the COVID-19 pandemic, i.e. between January 1, 2019 and January 31, 2020.

### Non-cancer control population

Normative data on quality of life, symptoms, functioning and comorbidities from a representative sample of the Dutch population was available from the PROFILES registry, and collected between May 4-26, 2020 [3]. This non-cancer control population was matched to the PLCRC population based on a frequency distribution over age (<30, 30-49, 50-69, ≥70) and sex strata to maximize the number of control participants matched to patients (ratio patients to norm 1:0.4).

### Data collection

Sociodemographic and clinical data were obtained from the NCR and include age, sex, date of primary cancer diagnosis, tumor location, stage at diagnosis, primary treatment received, and time since diagnosis (categorized). International Classification of Diseases (ICD)-10 was used to classify tumor types, and TNM was used to classify disease stage [4, 5]. Additional patient-reported sociodemographic data included educational level, marital status/partnership, living situation, height and weight. Patient-reported clinical variables included current presence of metastases, current treatment status, and presence of comorbidities (adapted Self-administered Comorbidity Questionnaire [6]). Detailed information on data categorization and design of questionnaires can be found in the **Supplementary Methods**.

***COVID-19-specific questionnaire (only during COVID-19).*** Questions regarding SARS-CoV-2 infection status, the fear of getting COVID-19, and the impact on cancer treatment and follow-up care as experienced by the patient were developed by the Dutch Federation of Cancer Patients Organisations (NFK) and the PROFILES Registry Group. Experiences with TC/VCs were assessed based on questions developed by Barsom et al. [7], while the EORTC QLQ-C30 response format was applied.

***HRQoL questionnaire (pre- and during COVID-19).*** The validated EORTC QLQ-C30 [8] was used to evaluate the patients' quality of life, daily functioning, and complaints of common symptoms. Only during COVID-19, an additional single-item scale was added to assess worries about future health (from EORTC library [9]). Linear transformation was used to obtain scores ranging from 0 to 100, where a higher score represents a higher ('better') level of functioning or a higher ('worse') level of symptoms [10].

***HADS questionnaire (pre- and during COVID-19).*** Anxiety and depressive symptoms of patients were assessed using the Hospital Anxiety and Depression Scale (HADS) [11]. To identify a clinically relevant level of anxiety or depression, a cut-off score of 8 was used [12].

***Loneliness questionnaire (only during COVID-19).*** Loneliness was assessed using the De Jong Gierveld short scales for social and emotional loneliness [13].

**Statistical analyses**

Patient characteristics were compared with the matched non-cancer control population by means of the independent samples t-test or Chi-square test. To facilitate the interpretation of cross-sectional HRQoL measures, prevalences of patients and control participants exceeding thresholds for clinical importance (TCIs) were compared [14]. General Linear Models, logistic- and ordinal regression models were used to compare well-being of CRC patients with the matched control population, while adjusting for education level, marital status, living situation, comorbidities, and COVID-19 status. Mean changes in HRQoL, anxiety and depression from <1 year pre- to during the COVID-19 pandemic were analyzed using the paired samples t-test. In the subgroup of patients in treatment, a sensitivity analysis was performed by metastatic vs. non-metastatic disease. Logistic regression was used to test differences in prevalence of patients reporting a clinically meaningful worsening of HRQoL (i.e. ≥10 points [15]), anxiety, and depression (i.e. ≥1.5 points [16]) by alterations in cancer care planning, while adjusting for age, sex, and comorbidities. Here, the Holm's sequential Bonferroni procedure was used to deal with inflated familywise error rates due to multiplicity [17]. Lastly, multivariable linear regression models including *a-priori* selected variables were used to determine factors associated with changes in global quality of life, anxiety, and depression from pre- to during the COVID-19 outbreak. A P value of less than .05 was considered statistically significant unless otherwise stated and all tests were 2-sided.

## Results

### Respondent characteristics

A total of 3,247 patients (65.1%) returned their COVID-19-specific questionnaire (**Figure 1**). A total of 2,664 non-cancer control participants (75.9%) completed the COVID-19-specific questionnaire, of whom 1,114 were age- and sex-matched to the patients.

Participants had a mean age of 67 (SD=10) years, 63.1% were male, and 19.7% of the patients reported presence of metastases (**Tables 1 and 2**). Patients reported more comorbidities, were higher educated, more often married, and more often lived with a partner without children, compared to the control population. Changes in hospital visits or treatment plan were associated with presence of comorbidities (respectively 37.0% and 41.5% with ≥2 comorbidities vs. 33.4% in the total CRC patient population).

### Changes in treatment and follow-up care during COVID-19

In our total study population, seventeen percent of patients had cancelled and/or postponed hospital visits, or visits that were changed into a telephone consultation (TV) or video consultation (VC), and 5.3% reported adjusted, postponed, and/or cancelled treatment.

Of 594 patients prior to or currently in treatment during COVID-19, 86 patients (14.5%) reported postponed and/or cancelled hospital visits, and 128

patients (21.5%) had their visit changed to a TC/VC (**Supplementary Table 1**). In 34 patients (5.7%) treatment was adjusted, in 48 patients (8.1%) postponed, and in 4 patients (0.7%) systemic or targeted treatment was cancelled. In case of physical complaints or concerns, patients reported a barrier to contact their general practitioner (11.3%) or medical specialist/nurse (6.4%). These undesired hesitations were even more common in the non-cancer control population (respectively 37.9% and 31.3%).

Of 2,456 patients with completed treatment, 363 patients (14.8%) had a hospital follow-up visit postponed and/or cancelled, 259 patients (10.5%) had a visit changed to a TC/VC (**Supplementary Table 1**). In case of physical complaints or concerns, these patients reported they contact their general practitioner (18.9%) or medical specialist/nurse (14.0%) less quickly as compared to pre-COVID-19, while respectively 70.8% and 60.2% reported no difference as compared to pre-COVID-19.

For 379 patients (12.2%), a face-to-face visit was changed into a TC (n=365) or VC (n=14). Most patients reported this change to be very/quite suitable (66.6%), but 47.1% reported that their physical condition could not/poorly be assessed (**Figure 2**). The majority (87.8%) was not worried about privacy issues, and although 44.9% would use a TC/VC again, a face-to-face consultation remained most preferred (70.4%), also in the subgroup of patients prior to or in treatment (80.7%).

**HRQoL and mental well-being in CRC patients vs. controls during COVID-19**

Although on average, CRC patients did not exceed TCIs regarding HRQoL during the COVID-19 pandemic, a statistically significantly larger proportion of CRC patients reported levels of fatigue, sleep disturbance, and functional impairments that exceed TCIs, as compared to the non-cancer control population (**Table 3**). Elevated levels of anxiety and depression were equally present in CRC patients and controls (anxiety: respectively 11.3% vs. 10.2%, depression: respectively 11.4% vs. 12.3%), but patients reported statistically significantly less moderate-severe loneliness - especially in terms of social loneliness (respectively 21.2% vs. 32.9%). Regarding concerns about getting SARS-CoV-2 infection, CRC patients were equally concerned as control participants.

**Changes in HRQoL, and mental well-being from pre- to during COVID-19**

Patients in follow-up (**Table 4**) who reported to have had changed/postponed/cancelled hospital visits, compared to patients without changes in cancer care planning, more frequently reported a clinically meaningful decrease in role (respectively 20.8% vs. 16.4%), emotional (respectively 18.7% vs. 10.3%), and social functioning (respectively 15.1% vs. 11.1%), and more often an increase in fatigue (respectively 27.2% vs. 18.1%), sleep disturbance (respectively 17.7% vs. 12.1%), and anxiety (respectively 25.1% vs. 18.9%).

In patients in treatment (**Table 5**), clinically meaningful worsening of HRQoL, anxiety and depression was more prevalent compared to patients in follow-up. In those with adjusted/postponed/cancelled treatment, compared to patients without changes in cancer care planning, clinically meaningful deterioration occurred most for physical (respectively 29.0% vs. 23.4%), cognitive (respectively 30.4% vs. 23.7%), and social functioning (respectively 34.8% vs. 28.8%), as well as for anxiety (respectively 29.5% vs. 22.1%). Sensitivity analysis within this group showed no statistically significant differences in HRQoL, depression, and anxiety between patients with metastatic vs. non-metastatic disease in neither of the three settings of cancer care planning (data not shown).

**Factors related to changes in HRQoL, anxiety and depression from pre- to during COVID-19**

Being prior to or in treatment during the COVID-19 pandemic, and being quite a bit/very worried about getting a SARS-CoV-2 infection were statistically significantly associated with a decrease in HRQoL (**Supplementary Table 2**). Factors statistically significantly associated with an increase in anxiety levels were increasing age (0.01 [95%CI = 0.00, 0.03], P = 0.03) and being quite a bit/very worried about a SARS-CoV-2 infection (1.0 [95%CI = 0.7, 1.3], P < 0.001) while having a partner but no children (-0.6 [95%CI = -1.1, -0.1], P = 0.02) was statistically significantly associated with lower anxiety levels. Factors associated with an increase in depression levels included increasing age (0.02 [95%CI = 0.01, 0.03], P = 0.007), being female (0.2 [95%CI = 0.0, 0.4], P = 0.04), being prior to or in treatment during the COVID-19 pandemic (0.6 [95%CI = 0.3, 1.0], P = 0.001), and being quite a bit/very worried about a SARS-CoV-2 infection (0.7 [95%CI: 0.5, 0.9], P <0.001), while having metastatic disease (-0.4 [95%CI = -0.8, -0.1], P = 0.001) was statistically significantly associated with lower depression levels.

### Discussion

During the first wave of SARS-CoV-2 infections in the Netherlands, hospital visits for patients with CRC were postponed or cancelled in 14.5% of patients in treatment, and in 14.8% of patients in follow-up. Hospital visits were changed into a TC/VC for 21.5% of patients in treatment, and in 10.5% of patients in follow-up. Although most patients reported this change to be very much/quite suitable, the vast majority favors a face-to-face consultation in the future. This pandemic has catalyzed the implementation of telemedicine to deliver healthcare, but remaining challenges both for patients and medical staff require continued research and development to ultimately find implementation in routine healthcare [18, 19]. Moreover, anticancer treatments were adjusted in 5.7%, postponed in 8.1%, and cancelled in 0.7% of the patients in treatment. During this early phase of the COVID-19 outbreak, a statistically significantly larger proportion of CRC patients reported levels of fatigue, sleep disturbance, and health-related functional impairments exceeding TCIs, compared to non-cancer controls. Mild/severe anxiety and depression were reported equally, while patients reported statistically significantly less social loneliness.

In patients in follow-up during the COVID-19 pandemic who had hospital visits cancelled/postponed/changed into TC/VC, the proportion of clinically meaningful worsening of role, emotional, and social functioning, fatigue, sleep disturbance and anxiety was statistically significantly higher compared to patients without changes in cancer care planning. Interestingly, in patients who were prior to or in treatment during the COVID-19 pandemic, the differences between patients with and without changes in treatment were non-statistically significant. Nevertheless, in this group, the overall prevalence of clinically meaningfully deteriorated HRQoL and anxiety was higher compared to those in follow-up. Despite the absence of statistical significance, patients of whom treatment was adjusted/postponed/cancelled, reported more often a clinically meaningful increase in anxiety than patients without changes.

Other studies that investigated anxiety and depression in cancer patients during the COVID-19 outbreak, reported mild-severe anxiety levels in 36-56% of patients, and mild-severe depression levels in 19-47% of patients [20-23]. This difference with our population (11.3%) might be explained by differences in study population, cancer types, and nationality. In a Dutch study in breast cancer patients, comparable mean levels of anxiety and depression were observed that also did not statistically significantly change from pre-COVID-19 to during the COVID-19 pandemic [24]. Our results confirm previously reported data of two large Dutch cohort studies that reported on practice patterns and perceived HRQoL in patients with cancer during the COVID-19 outbreak [24, 25]. First, Bargon *et al.* previously reported statistically significant but minimal changes in HRQoL scores from before to during COVID-19 in breast cancer patients and survivors [24]. Second, van de Poll-Franse *et al.*, who included part of the current PLCRC data, reported no clinically meaningful differences in HRQoL scales between patients with various tumor types and a matched control population [25]. The current analysis adds to these findings by showing that despite small average changes in HRQoL, a large proportion of CRC patients does experience health-related functional impairments, and fatigue and sleep disturbance beyond thresholds for TCI – statistically significantly more than the age and sex-matched non-cancer control population. Additionally, we showed that changes in cancer care planning are associated with an increased prevalence of clinically meaningful deterioration of daily functioning, fatigue, sleep disturbance, and anxiety, that are as such perceptible to patients.

A positive finding was that, compared to the control population, CRC patients experienced statistically significantly less social loneliness. Possibly, the government's measures such as restricted freedom of movement and social distancing have less impact on cancer patients as they may already be much more cautious about infections. To some, the COVID-19 crisis may have distracted from focusing on their disease which, together with existing or increased social support from friends and family, could have lessened the impact on psychosocial well-being.

Altogether, we emphasize that despite minimal average changes in HRQoL, anxiety and depression from pre-COVID to during the COVID-19 pandemic, substantial differences do occur on an individual patient level. Therefore, individual- and cancer-specific factors related to changes in HRQoL and mental well-being were identified. Age, sex, having a partner but no children, metastatic disease, being in treatment, and being quite a bit/very much worried about getting a SARS-CoV-2

infection were factors statistically significantly associated with changes in global quality of life, anxiety or depression. Although individual factors are only associated with small changes, they might be intertwined and could cumulatively lead to meaningful changes. These factors should be taken into consideration by healthcare professionals and used to identify patients at risk of deterioration of HRQoL and mental well-being during subsequent waves of SARS-CoV-2 infections or comparable future pandemics. Especially in the group of patients presenting with one or more of the above mentioned factors, discussing (mental) health status and possible support is recommended.

A limitation of the current study was that some clinical information were self-reported by patients, which could have led to misclassification and therefore dilution of our results. Although expected to be of little influence [26], results may be prone to seasonality, as questionnaires were completed during spring 2020 and compared to 2019 year-round. In contrast, PROs are highly valuable as a direct measure of how patients' health and well-being were perceived. Second, the small number of patients in treatment with changes in cancer care might have influenced the magnitude of statistical significance. Therefore, it is important to consider the direction and size of the observed effect sizes. Third, since we lack information on reasons to not participate in this study, an under- or overestimation of the results due to selective response could not be ruled out. Lastly, we observed baseline differences between CRC patients and the control population, which could influence comparisons between the two groups, however, models were adjusted for the imbalanced factors.

There are also several strengths related to this analysis, including the large number of respondents which allowed for comprehensive analyses in a specific patient population. Moreover, the benefit of implementing this questionnaire-based study in an ongoing cohort with clinical data and PROs from diagnosis onwards is that data before the COVID-19 outbreak was available allowing for a comparison with the year before. Additionally, this enables longitudinal modelling of repeated PROs during future SARS-CoV-2 infection waves and subsequent government-imposed national prevention measures. This will provide further insight in the impact of ongoing social distancing and other nationwide prevention measures on patients' well-being and will help tailor mental support strategies.

To conclude, between 9.8%-18.9% of patients without changes in cancer care during follow-up reported clinically meaningful worsening of HRQoL, anxiety and depression compared to the year before the COVID-19 outbreak, and in patients prior to or in treatment, these prevalences increased to 14.6%-34.9%. Both in patients in treatment and in follow-up, changes in cancer care planning were associated with increased deterioration of several aspects of HRQoL and anxiety. Interestingly, during COVID-19, patients experienced statistically significantly less social loneliness than the non-cancer control population. To optimally meet patient needs, (mental) health status and possible support options should be discussed - especially in patients presenting with one or more risk factors - during future SARS-CoV-2 infection waves or comparable pandemics.

### Funding

The current work was not supported by a specific funding source.

### Notes

**Role of the funders:** Not applicable.

**Disclosures:** MK reports institutional financial interests with Amgen, Bayer, BMS, Merck-Serono, Nordic Pharma, Roche, Servier, Sirtex, and Sanofi-Aventis. MK reports the following non-financial interests: an advisory role for ZON-MW, membership of the scientific board of the Dutch Cancer Society (KWF), chairmanship of the Dutch Colorectal Cancer Group (DCCG), principal investigator (PI) of the Prospective Dutch Colorectal Cancer (PLCRC) cohort. GRV reports institutional financial interests with Servier, Merck, Bayer, Sirtex, BMS, and Lilly. All other authors report no competing interests.

**Acknowledgements:** Full list of consortium members and their affiliations for "the PLCRC study group": Geerard L. Beets (Department of Surgery, Nederlands Kanker Instituut - Antoni van Leeuwenhoek Hospital, Amsterdam, The Netherlands), Eric J.Th. Belt (Department of Surgery, Albert Schweitzer Hospital, Dordrecht, The Netherlands), Maaike Berbée (Department of Radiotherapy, Maastro Clinic, Maastricht, The Netherlands), Frederique H. Beverdam (Department of Surgery, Franciscus Gasthuis & Vlietland Hospital, Schiedam, The Netherlands), Ruud Blankenburgh (Department of Medical Oncology, Saxenburgh Hospital, Hardenberg, The Netherlands), Peter Paul L.O. Coene (Department of Surgery, Maasstad Hospital,

Rotterdam, The Netherlands), Hester van Cruijsen (Department of Medical Oncology, Antonius Hospital, Sneek, The Netherlands), Jan Willem T. Dekker (Department of Surgery, Reinier de Graaf Hospital, Delft, The Netherlands), Joyce M. van Dodewaard-de Jong (Department of Medical Oncology, Meander Medical Center, Amersfoort, The Netherlands), Frans L.G. Erdkamp (Department of Medical Oncology, Zuyderland Hospital, Heerlen, The Netherlands), Jan Willem B. de Groot (Department of Medical Oncology, Isala Oncology Center, Zwolle, The Netherlands), Annebeth W. Haringhuizen (Department of Medical Oncology, Ziekenhuis Gelderse Vallei, Ede, The Netherlands), Helgi H. Helgason (Department of Medical Oncology, Haaglanden Medical Center, Den Haag, The Netherlands), Mathijs P. Hendriks (Department of Medical Oncology, Northwest Clinics, Alkmaar, The Netherlands), Ignace H.J.T. de Hingh (Department of Surgery, Catharina Hospital, Eindhoven, The Netherlands), Ronald Hoekstra (Department of Medical Oncology, Ziekenhuisgroep Twente, Hengelo, The Netherlands), Jan N.M. Ijzermans (Department of Surgery, Erasmus MC, University Medical Center Rotterdam, Rotterdam, The Netherlands), Jan Jansen (Department of Surgery, Admiraal de Ruyter Hospital, Goes, The Netherlands), Frank W.H. Kloppenberg (Department of Surgery, Treant Hospital, Emmen, The Netherlands), Anja U.G. van Lent (Department of Gastroenterology and Hepatology, Onze Lieve Vrouwe Hospital, Amsterdam, The Netherlands), Maartje Los (Department of Medical Oncology, St. Antonius Hospital, Nieuwegein, The Netherlands), Martijn R. Meijerink (Department of Radiology & Nuclear Medicine, Amsterdam University Medical Center - loc. VU, University of Amsterdam, Amsterdam, The Netherlands),

Leonie J.M. Mekenkamp (Department of Medical Oncology, Medisch Spectrum Twente, Enschede, The Netherlands), Peter Nieboer (Department of Medical Oncology, Wilhelmina Hospital, Assen, The Netherlands), Koen C.M.J. Peeters (Department of Surgery, Leiden University Medical Center, University of Leiden, Leiden, The Netherlands), Natascha A.J.B. Peters (Department of Medical Oncology, Sint Jans Hospital, Weert, The Netherlands), Marco B. Polée (Department of Medical Oncology, Medical Center Leeuwarden, Leeuwarden, The Netherlands), Johannes F.M. Pruijt (Department of Medical Oncology, Jeroen Bosch Hospital, Den Bosch, The Netherlands), Patricia Quarles van Ufford-Mannesse (Department of Medical Oncology, Haga Hospital, Den Haag, The Netherlands), Ron C. Rietbroek (Department of Medical Oncology, Rode Kruis Hospital, Beverwijk, The Netherlands), Anandi H.W. Schiphorst (Department of Surgery, Diakonessenhuis Hospital, Utrecht, The Netherlands), Arjan Schouten van der Velden (Department of Surgery, St Jansdal Hospital, Harderwijk, The Netherlands), Ruud W.M. Schrauwen (Department of Gastroenterology and Hepatology, Bernhoven Hospital, Uden, The Netherlands), Mark P.S. Sie (Department of Medical Oncology, ZorgSaam Hospital, Terneuzen, The Netherlands), Lieke Simkens (Department of Medical Oncology, Maxima Medical Center, Eindhoven, The Netherlands), Dirkje W. Sommeijer (Department of Medical Oncology, Flevo Hospital, Almere, The Netherlands, and Department of Medical Oncology, Amsterdam University Medical Center - loc. AMC, University of Amsterdam, Amsterdam, The Netherlands), Dirk J.A. Sonneveld (Department of Surgery, Dijklander Hospital, Purmerend, The Netherlands), Leontine E.A. Spierings

(Department of Medical Oncology, Alrijne Hospital, Leiderdorp, The Netherlands), Hein B.A.C. Stockmann (Department of Surgery, Spaarne Hospital, Haarlem, The Netherlands), Koen Talsma (Department of Surgery, Deventer Hospital, Deventer, The Netherlands), Frederiek Terheggen (Department of Medical Oncology, Bravis Hospital, Roosendaal, The Netherlands), Albert J. ten Tije (Department of Medical Oncology, Amphia Hospital, Breda, The Netherlands), Manuel L.R. Tjin-A-Ton (Department of Medical Oncology, Rivierenland Hospital, Tiel, The Netherlands), Renzo P. Veenstra (Department of Gastroenterology and Hepatology, Martini Hospital, Groningen, The Netherlands), Ankie M.T. van der Velden (Department of Medical Oncology, Tergooi Hospital, Hilversum, The Netherlands), Maarten Vermaas (Department of Surgery, Ijsselland Hospital, Capelle aan den Ijssel, The Netherlands), Wouter J. Vles (Department of Surgery, Ikazia Hospital, Rotterdam, The Netherlands), F. Jeroen Vogelaar (Department of Surgery, Viecuri Hospital, Venlo, The Netherlands), Theo van Voorthuizen (Department of Medical Oncology, Rijnstate Hospital, Arnhem, The Netherlands), Aad I. de Vos (Department of Medical Oncology, Van Weel-Bethesda Hospital, Dirksland, The Netherlands), Judith de Vos-Geelen (Department of Medical Oncology, Maastricht University Medical Center, Maastricht, The Netherlands), Johannes A. Wegdam (Department of Surgery, Elkerliek Hospital, Helmond, The Netherlands), Johannes H.W. de Wilt (Department of Surgery, Radboud University Medical Center, University of Nijmegen, Nijmegen, The Netherlands), David D.E. Zimmerman (Department of Surgery, Elisabeth-TweeSteden Hospital, Tilburg, The Netherlands).

The Prospective Dutch Colorectal Cancer (PLCRC) cohort is an initiative of the Dutch Colorectal Cancer Group (DCCG). PLCRC is supported by the Dutch Cancer Society; Stand Up to Cancer; ZonMw; Health Holland; Maag Lever Darm Stichting; Lilly (unrestricted grant); Merck (unrestricted grant); Bristol-Myers Squibb (unrestricted grant); Bayer (unrestricted grant); and Servier (unrestricted grant).

We would like to thank all patients who participated in this study.

**Author contributions:** Jeroen W.G. Derksen: Conceptualization, Methodology, Data curation, Formal analysis, Visualization, Writing - original draft. Anne M. May: Conceptualization, Methodology, Supervision, Writing - review & editing. Lonneke V. van de Poll-Franse: Conceptualization, Writing - review & editing. Belle H. de Rooij: Data curation, Formal analysis, Writing - review & editing. Dorothee Hafkenscheid: Writing - review & editing. Helena M. Verkooijen: Funding acquisition, Writing - review & editing. Miriam Koopman: Funding acquisition, Writing - review & editing. Geraldine R. Vink: Funding acquisition, Conceptualization, Supervision, Writing - review & editing.

### Data Availability

The data underlying this article were provided by the Netherlands Comprehensive Cancer Organisation (IKNL) and the Dutch Colorectal Cancer Group (DCCG) under license. Data will be shared on request to the corresponding author with permission of IKNL and the scientific board of the Prospective Dutch Colorectal Cancer Cohort/DCCG.

### Tables

**Table 1.** Sociodemographic, comorbidity and COVID-19 characteristics of CRC patients (N=3,247), subgroups according to changes in treatment or hospital visits, and the age- and sex- matched non-cancer control population (N=1,114)

| Characteristics | Total CRC study population | Patients without changes in treatment or visits [c] | Patients with changes in hospital visits | Patients with changes in treatment | *P* [d] | Matched control population | *P* [e] |
|---|---|---|---|---|---|---|---|
| Total No. | 3,247 | 2,429 | 551 | 171 | | 1,114 | |
| Mean age (SD), y | 67.1 (10.1) | 67.3 (9.9) | 66.1 (10.2) | 66.9 (11.6) | 0.06 | 66.7 (12.0) | 0.31 |
| ≥70 years, No. (%) | 1406 (43.3) | 1,057 (43.6) | 218 (39.7) | 77 (45.0) | 0.22 | 482 (43.3) | 0.98 |
| Sex (men), No. (%) | 2049 (63.1) | 1,547 (63.7) | 342 (62.1) | 107 (62.6) | 0.76 | 703 (63.1) | 1.00 |
| Education level [a], No. (%) | | | | | 0.31 | | <0.001 |
| Low | 913 (28.3) | 702 (29.0) | 142 (26.1) | 40 (23.5) | | 458 (41.2) | |
| Medium | 977 (30.3) | 723 (29.9) | 176 (32.3) | 51 (30.0) | | 246 (22.1) | |
| High | 1,336 (41.4) | 992 (41.0) | 227 (41.7) | 79 (46.5) | | 407 (36.6) | |
| Marital status (partner), No. (%) | 2,638 (81.7) | 1,982 (81.9) | 441 (81.1) | 137 (81.1) | 0.88 | 753 (67.6) | <0.001 |
| Living situation, No. (%) | | | | | 0.08 | | <0.001 |
| Alone | 575 (17.8) | 434 (17.9) | 101 (18.5) | 30 (17.5) | | 285 (25.6) | |
| Partner, no children | 2,187 (67.5) | 1,658 (68.3) | 365 (66.8) | 104 (60.8) | | 577 (51.8) | |
| Partner and children | 267 (8.2) | 193 (8.0) | 40 (7.3) | 23 (13.5) | | 176 (15.8) | |
| No partner, with children | 140 (4.3) | 99 (4.1) | 22 (4.0) | 10 (5.8) | | 28 (2.5) | |
| Other | 69 (2.1) | 43 (1.8) | 18 (3.3) | 4 (2.3) | | 48 (4.3) | |
| Mean BMI (SD), kg/m$^2$ | 26.5 (4.3) | 26.5 (4.3) | 26.4 (4.1) | 26.5 (5.4) | 0.85 | 26.4 (4.4) | 0.41 |
| BMI, No. (%) | | | | | | | |
| <25 kg/m$^2$ | 1,296 (40.2) | 962 (39.8) | 226 (41.4) | 75 (44.4) | 0.66 | 467 (42.0) | 0.56 |
| 25-<30 kg/m$^2$ | 1,366 (42.4) | 1,030 (42.7) | 230 (42.1) | 63 (37.3) | | 455 (41.0) | |

| | | | | | | | |
|---|---|---|---|---|---|---|---|
| ≥30 kg/m$^2$ | 561 (17.4) | 423 (17.5) | 90 (16.5) | 31 (18.3) | | 189 (17.0) | |
| Comorbidities, No. (%) | | | | | 0.02 | | <0.001 |
| No | 1,184 (36.5) | 914 (37.6) | 177 (32.1) | 54 (31.6) | | 345 (31.0) | |
| 1 | 977 (30.1) | 728 (30.0) | 170 (30.9) | 46 (26.9) | | 312 (28.0) | |
| ≥2 | 1,086 (33.4) | 787 (32.4) | 204 (37.0) | 71 (41.5) | | 457 (41.0) | |
| SARS-CoV-2 virus infection, No. (%) | | | | | 0.17 | | 0.004 |
| Yes, tested positive | 9 (0.3) | 5 (0.2) | 1 (0.2) | 2 (1.2) | | 2 (0.2) | |
| Probably, symptoms [b] and/or fever | 182 (5.7) | 136 (5.6) | 36 (6.5) | 7 (4.1) | | 67 (6.0) | |
| No, tested negative | 84 (2.6) | 62 (2.6) | 14 (2.5) | 7 (4.1) | | 9 (0.8) | |
| No, not tested and no symptoms | 2,944 (91.5) | 2,217 (91.6) | 499 (90.7) | 153 (90.5) | | 1,036 (93.0) | |

[a] low = secondary education (high school) or lower, medium = secondary (vocational) education, high = higher (vocational) education/university. BMI = body mass index; SARS-CoV-2 = Severe Acute Respiratory Syndrome coronavirus 2.

[b] respiratory symptoms and/or fever (38 degrees Celsius or higher) and/or 2 or more symptoms of: flu or sickness, muscle pain, eye pain or headache, and/or I have been in contact with someone who is (had been) infected with COVID-19. Missing: education level (n=21 patients, n=3 controls), living situation (n=9 patients), BMI (n=24 patients, n=3 controls), SARS-CoV-2 infection (n=28 patients).

[c] Missing: data on changes in care planning (N=96 patients). Percentages do not always add up to 100 because of rounding to whole numbers.

[d] P values for comparisons by changes in treatment or hospital visits using one-way ANOVA and Chi-square test.

[e] P values for comparison between total CRC study population and the matched control population using independent samples t-test and Chi-square test.

**Table 2.** Disease and treatment characteristics of CRC patients (N=3,247)

| Characteristics | Total CRC study population | Patients without changes in treatment or visits [c] | Patients with changes in hospital visits | Patients with changes in treatment | *P* [d] |
|---|---|---|---|---|---|
| Total No. (%) | 3,247 | 2,429 (75) | 551 (17) | 171 (5) | |
| Tumor type, No. (%) | | | | | 0.45 |
| Colon (C18.0-18.9) | 1,448 (44.6) | 1,092 (45.0) | 232 (42.1) | 79 (46.2) | |
| Rectum (C19.9, C20.9) | 1,047 (32.2) | 793 (32.6) | 176 (31.9) | 52 (30.4) | |
| Unknown [a] | 752 (23.2) | 544 (22.4) | 143 (26.0) | 40 (23.4) | |
| Tumor stage at diagnosis, No. (%) | | | | | |
| Stage I | 516 (15.9) | 397 (16.3) | 85 (15.4) | 16 (9.4) | <0.001 |
| Stage II | 583 (18.0) | 449 (18.5) | 99 (18.0) | 17 (9.9) | |
| Stage III | 1,101 (33.9) | 833 (34.3) | 178 (32.3) | 65 (38.0) | |
| Stage IV | 286 (8.8) | 199 (8.2) | 44 (8.0) | 33 (19.3) | |
| Not applicable | 9 (0.3) | 7 (0.3) | 2 (0.4) | 0 (0) | |
| Unknown [a] | 752 (23.2) | 544 (22.4) | 143 (26.0) | 40 (23.4) | |
| Time since primary diagnosis, No. (%) | | | | | 0.12 |
| <6 months | 313 (9.6) | 225 (9.3) | 62 (11.3) | 14 (8.2) | |
| 6-<12 months | 568 (17.5) | 407 (16.8) | 108 (19.6) | 34 (19.9) | |
| 12-<24 months | 955 (29.4) | 702 (28.9) | 175 (31.8) | 52 (30.4) | |
| 24-<60 months | 1,085 (33.4) | 844 (34.7) | 159 (28.9) | 49 (28.7) | |
| ≥60 months | 250 (7.7) | 195 (8.0) | 35 (6.4) | 16 (9.4) | |
| Unknown [a] | 76 (2.3) | 56 (2.3) | 12 (2.2) | 6 (3.5) | |
| Current presence of metastases [b], No. (%) | | | | | <0.001 |
| Yes | 640 (19.7) | 438 (19.5) | 102 (19.0) | 83 (49.4) | |

| | | | | | |
|---|---|---|---|---|---|
| No | 2,119 (65.3) | 1,627 (72.5) | 365 (68.1) | 66 (39.3) | |
| Don't know | 271 (8.3) | 179 (8.0) | 69 (12.9) | 19 (11.3) | |
| Current phase of disease/treatment [b], No. (%) | | | | | <0.001 |
| Still have to start Tx | 27 (0.8) | 15 (0.6) | 6 (1.1) | 6 (3.5) | |
| Currently being treated | 567 (17.5) | 380 (15.6) | 93 (16.9) | 79 (46.2) | |
| Completed Tx, currently in follow-up care | 2,456 (75.6) | 1,859 (76.5) | 445 (80.8) | 84 (49.1) | |
| Completed Tx, no follow-up care | 181 (5.6) | 166 (6.8) | 5 (0.9) | 2 (1.2) | |
| Unknown | 16 (0.5) | 9 (0.4) | 2 (0.4) | 0 (0) | |
| Current treatment, or soon to be started [b], No. (%) | | | | | |
| Chemotherapy | 371 (11.4) | 240 (10.4) | 63 (11.5) | 61 (35.7) | <0.001 |
| Surgery | 141 (4.3) | 104 (4.5) | 24 (4.4) | 9 (5.3) | 0.89 |
| Immunotherapy | 81 (2.5) | 52 (2.3) | 15 (2.7) | 12 (7.0) | 0.001 |
| Radiotherapy | 48 (1.5) | 38 (1.6) | 4 (0.7) | 5 (2.9) | 0.10 |
| Targeted therapy | 27 (0.8) | 19 (0.8) | 1 (0.2) | 6 (3.5) | <0.001 |
| Symptom management | 66 (2.0) | 33 (1.4) | 20 (3.7) | 10 (5.8) | <0.001 |
| Active surveillance/wait and see | 268 (8.3) | 189 (8.2) | 44 (8.1) | 28 (16.4) | 0.001 |
| Other | 328 (10.1) | 217 (9.4) | 60 (11.0) | 37 (21.6) | <0.001 |
| Current supportive care [b], No. (%) | | | | | |
| Oncology nurse | 509 (15.7) | 361 (14.9) | 97 (17.6) | 39 (22.8) | 0.01 |
| General Practitioner | 258 (7.9) | 172 (7.1) | 55 (10.0) | 24 (14.0) | 0.001 |
| Physical therapy | 177 (5.5) | 114 (4.7) | 38 (6.9) | 22 (12.9) | <0.001 |
| Psychological care | 122 (3.8) | 68 (2.8) | 33 (6.0) | 16 (9.4) | <0.001 |
| Dietician | 103 (3.2) | 76 (3.1) | 21 (3.8) | 6 (3.5) | 0.71 |
| Oncological Rehabilitation | 67 (2.1) | 37 (1.5) | 16 (2.9) | 9 (5.3) | 0.001 |
| Other | 391 (12.0) | 249 (10.3) | 92 (16.7) | 32 (18.7) | <0.001 |

[a] Unknown because clinical data was not yet available due to the time-lag within the NCR. Percentages do not always add up to 100 because of rounding to whole numbers. CRC = colorectal cancer; Tx = treatment.

[b] Patient-reported data from COVID-19-specific questionnaire. Other supportive care: patient support groups, religious/spiritual care, sexologist, and creative therapy.

[c] Missing: data on changes in care planning (n=96 patients).

[d] P values for comparisons by changes in treatment or hospital visits using Chi-square test.

**Table 3.** Quality of life (incl. daily functioning, fatigue and sleep disturbances), anxiety, depression and loneliness as reported by patients with CRC (N=3,247), compared to the matched non-cancer control population during the COVID-19 pandemic (N=1,114).

| Patient-reported outcome | Colorectal cancer patients [b] | Matched control population | *P* [c] |
|---|---|---|---|
| Total No. | 3,247 | 1,114 | |
| HRQoL - EORTC QLQ-C30 (0-100), mean (SD) | | | |
| Global quality of life | 76.7 (18) | 74.2 (17) | 0.04 |
| Physical functioning | 86.4 (17) | 89.6 (15) | <0.001 |
| Role functioning | 82.5 (25) | 88.8 (21) | <0.001 |
| Emotional functioning | 86.5 (17) | 87.9 (16) | <0.001 |
| Cognitive functioning | 88.3 (17) | 92.6 (14) | <0.001 |
| Social functioning | 88.2 (20) | 95.0 (14) | <0.001 |
| Fatigue | 20.3 (22) | 14.7 (19) | <0.001 |
| Sleep disturbances | 17.6 (25) | 14.8 (24) | <0.001 |
| Future health worries | 27.2 (25) | 21.6 (22) | <0.001 |
| Prevalence exceeding TCI [a], No. (%) | | | |
| Physical functioning (<83) | 963 (29.8) | 245 (22.0) | <0.001 |
| Role functioning (<58) | 459 (14.2) | 96 (8.6) | <0.001 |
| Emotional functioning (<71) | 534 (16.5) | 158 (14.2) | 0.006 |
| Cognitive functioning (<75) | 609 (18.9) | 115 (10.3) | <0.001 |
| Social functioning (<58) | 286 (8.9) | 36 (3.2) | <0.001 |
| Fatigue (>39) | 503 (15.6) | 106 (9.5) | <0.001 |
| Sleep disturbances (>50) | 361 (11.2) | 102 (9.2) | 0.002 |
| HADS (0-21), mean (SD) | | | |
| Anxiety (0-21) | 3.4 (3) | 3.2 (3) | 0.05 |
| Depression (0-21) | 3.1 (3) | 3.2 (3) | 0.15 |
| Categorical, No. (%) | | | |
| Mild/severe anxiety (8-21) | 365 (11.3) | 114 (10.2) | 0.03 |
| Mild/severe depression (8-21) | 367 (11.4) | 137 (12.3) | 0.24 |
| Overall loneliness (0-6), mean (SD) | 1.6 (2) | 1.9 (2) | <0.001 |
| Categorical, No. (%) | | | 0.06 |
| No loneliness (0-1) | 1,831 (57.0) | 549 (49.3) | |
| Moderate loneliness (2-4) | 1,142 (35.6) | 444 (39.9) | |
| Severe loneliness (5-6) | 238 (7.4) | 121 (10.9) | |
| Emotional loneliness (0-3), mean (SD) | 0.9 (1) | 0.9 (1) | 0.45 |
| Categorical, No. (%) | | | 0.001 |
| No emotional loneliness (0-1) | 2,314 (72.4) | 819 (73.5) | |
| Emotionally lonely (2-3) | 880 (27.6) | 295 (26.5) | |
| Social loneliness (0-3), mean (SD) | 0.7 (1.1) | 1.0 (1.2) | <0.001 |

| | | | |
|---|---|---|---|
| Categorical, No. (%) | | | <0.001 |
| No social loneliness (0-1) | 2,514 (78.8) | 748 (67.1) | |
| Socially lonely (2-3) | 677 (21.2) | 366 (32.9) | |
| Worried about getting SARS-CoV-2 infection, No. (%) | | | 0.03 |
| Not at all | 590 (18.4) | 198 (17.8) | |
| A little | 1,935 (60.2) | 724 (65.1) | |
| Quite a bit | 573 (17.8) | 150 (13.5) | |
| Very much | 117 (3.6) | 40 (3.6) | |

[a] TCIs for the functioning and symptom scales of the EORTC QLQ-C30, i.e. below the TCI for functioning scales, or above the TCI for symptom scales indicate clinically important problems or symptoms. No TCI available for global quality of life and future health worries. HRQoL = Health-related quality of life; EORTC QLQ-C30 = European Organisation for Research and Treatment of Cancer Quality of Life Questionnaire C30; TCI = Thresholds for Clinical Importance; HADS = Hospital Anxiety and Depression Scale; SARS-CoV-2 = Severe Acute Respiratory Syndrome coronavirus 2.

[b] Missing: QLQ-C30 (n=15-45, 0.5-1.4%), HADS (n=17, 0.4%), loneliness (n=36, 0.8%), emotional loneliness (n=53, 1.2%), social loneliness (n=56, 1.3%), worried about SARS-CoV-2 infection (n=34, 0.8%). Percentages do not always add up to 100 because of rounding to whole numbers.

[c] P values for comparison between colorectal cancer patients and the matched control population using General Linear Models, ordinal and logistic regression models (2-sided), adjusted for education level, marital status, living situation, comorbidities, and COVID-19 status.

**Table 4.** Changes in quality of life, anxiety, and depression from <1 year pre- to during the COVID-19 pandemic in patients with CRC who completed treatment and were in follow-up, stratified for changes in cancer care planning

| | CRC patients without changes in treatment plan or hospital visits (n=1,786) | | | | CRC patients with hospital visits cancelled, postponed or changed into TC/VC (n=390) | | | | |
|---|---|---|---|---|---|---|---|---|---|
| Patient-reported outcome | Pre-COVID, Mean (SD) | During COVID, Mean (SD) | Within group change (95%CI) | Clinically meaningful worsening [a] No. (%) | Pre-COVID, Mean (SD) | During COVID, Mean (SD) | Within group change (95%CI) | Clinically meaningful worsening [a] No. (%) | *P* [b] |
| HRQoL (0-100) | | | | | | | | | |
| Global quality of life | 80.8 (16) | 80.4 (16) | -0.4 (-1.1, -0.3) | 312 (17.6) | 75.4 (18) | 74.9 (17) | -0.5 (-2.1, 1.0) | 74 (19.0) | 0.49 |
| Physical functioning | 88.7 (15) | 88.8 (15) | +0.2 (-0.3, 0.7) | 174 (9.8) | 86.8 (16) | 86.9 (16) | +0.1 (-1.2, 1.4) | 49 (12.6) | 0.19 |
| Role functioning | 84.8 (23) | 87.5 (21) | +2.7 (1.7, 3.8) | 293 (16.4) | 78.8 (26) | 81.8 (24) | +3.0 (0.5, 5.5) | 81 (20.8) | 0.03 |
| Emotional functioning | 87.7 (17) | 89.0 (15) | +1.3 (0.7, 2.0) | 183 (10.3) | 83.4 (19) | 83.0 (19) | -0.3 (-2.1, 1.6) | 73 (18.7) | <0.001[c] |
| Cognitive functioning | 88.2 (16) | 90.2 (16) | +2.0 (1.3, 2.6) | 254 (14.2) | 84.3 (19) | 86.5 (18) | +2.2 (0.6, 3.8) | 61 (15.6) | 0.61 |
| Social functioning | 88.8 (18) | 92.5 (18) | +3.7 (2.8, 4.5) | 198 (11.1) | 82.9 (22) | 87.6 (20) | +4.7 (2.5, 7.0) | 59 (15.1) | 0.03 |
| Fatigue | 19.9 (21) | 15.5 (19) | -4.4 (-5.3, -3.6) | 323 (18.1) | 26.2 (23) | 22.8 (24) | -3.3 (-5.5, -1.2) | 106 (27.2) | <0.001[c] |
| Sleep disturbances | 17.5 (24) | 14.4 (22) | -3.1 (-4.1, -2.1) | 215 (12.1) | 22.5 (27) | 20.2 (27) | -2.2 (-5.0, 0.6) | 69 (17.7) | 0.01 |
| HADS (0-21) | | | | | | | | | |
| Anxiety | 3.1 (3.1) | 3.0 (3.1) | -0.1 (-0.2, 0.0) | 289 (18.9) | 3.9 (3.6) | 4.0 (3.4) | 0.2 (-0.1, 0.5) | 85 (25.1) | 0.01 |
| Depression | 2.8 (3.1) | 2.6 (3.0) | -0.1 (-0.3, 0.0) | 247 (16.2) | 3.8 (3.7) | 3.6 (3.5) | -0.2 (-0.5, 0.1) | 54 (16.0) | 0.92 |

[a] i.e. ≥10 point decrease in HRQoL functional scales or ≥10 point increase in symptoms scales, and ≥1.5 points increase in HADS scales. COVID-19 = Coronavirus disease 2019; CRC = Colorectal cancer; TC/VC = Telephone consultation/Video consultation; HRQoL = Health-related quality of life; HADS = Hospital Anxiety and Depression Scale.

[b] P values for difference in prevalence of worsened HRQoL and HADS between groups, using logistic regression models (2-sided), adjusted for: age, sex, and number of comorbidities. Group numbers differ from Table 1 as these depend on the availability of pre-COVID questionnaires.

[c] Statistically significant after Holm's Sequential Bonferroni correction.

**Table 5.** Changes in quality of life, anxiety, and depression from <1 year pre- to during the COVID-19 pandemic in patients with CRC who were prior to or currently in treatment, stratified for changes in cancer care planning

| | CRC patients without changes in treatment plan or hospital visits (n=275) | | | | CRC patients with hospital visits cancelled, postponed or changed into TC/VC (n=74) | | | | | CRC patients with adjusted, postponed, or cancelled treatment (n=69) | | | | |
|---|---|---|---|---|---|---|---|---|---|---|---|---|---|---|
| Patient-reported outcome | Pre-COVID, Mean (SD) | During COVID, Mean (SD) | Within group change (95%CI) | Clinically meaningful worsening[a] No. (%) | Pre-COVID, Mean (SD) | During COVID, Mean (SD) | Within group change (95%CI) | Clinically meaningful worsening[a] No. (%) | *P* [b] | Pre-COVID, Mean (SD) | During COVID, Mean (SD) | Within group change (95%CI) | Clinically meaningful worsening[a] No. (%) | *P* [b] |
| HRQoL (0-100) | | | | | | | | | | | | | | |
| Global quality of life | 71.5 (19) | 66.9 (20) | -4.6 (-7.4, -1.9) | 95 (34.9) | 69.4 (17) | 66.4 (21) | -3.0 (-7.1, 1.1) | 19 (26.4) | 0.25 | 71.4 (22) | 66.4 (22) | -5.0 (-9.8, -0.1) | 21 (30.4) | 0.48 |
| Physical functioning | 84.8 (16) | 81.4 (18) | -3.4 (-5.3, -1.4) | 64 (23.4) | 81.4 (17) | 78.9 (19) | -2.5 (-5.4, 0.4) | 16 (21.6) | 0.96 | 81.4 (21) | 78.6 (20) | -2.7 (-6.5, 1.1) | 20 (29.0) | 0.34 |
| Role functioning | 72.4 (29) | 71.1 (29) | -1.3 (-5.1, 2.6) | 85 (30.9) | 70.9 (29) | 70.7 (31) | -0.2 (-7.2, 6.8) | 21 (28.4) | 0.97 | 68.8 (34) | 67.4 (32) | -1.4 (-8.7, 5.8) | 24 (34.8) | 0.68 |
| Emotional functioning | 81.4 (18) | 81.8 (19) | +0.5 (-1.6, 2.5) | 40 (14.6) | 81.5 (19) | 82.1 (19) | +0.6 (-3.3, 4.5) | 13 (17.8) | 0.35 | 72.3 (22) | 74.9 (22) | +2.5 (-2.3, 7.4) | 11 (15.9) | 0.86 |
| Cognitive functioning | 86.0 (17) | 84.9 (19) | -1.1 (-3.2, 1.0) | 65 (23.7) | 83.3 (17) | 85.2 (16) | +1.8 (-2.4, 6.0) | 17 (23.3) | 0.91 | 81.4 (21) | 80.9 (19) | -0.5 (-4.7, 3.7) | 21 (30.4) | 0.33 |
| Social functioning | 79.1 (23) | 78.6 (23) | -0.5 (-3.5, 2.5) | 79 (28.8) | 80.1 (25) | 76.9 (25) | -3.2 (-8.9, 2.5) | 23 (31.5) | 0.66 | 77.5 (25) | 74.6 (28) | -2.9 (-9.1, 3.3) | 24 (34.8) | 0.46 |
| Fatigue | 30.1 (23) | 30.0 (23) | -0.1 (-3.1, 3.0) | 89 (32.5) | 30.5 (23) | 31.2 (24) | +0.7 (-4.9, 6.2) | 26 (35.1) | 0.55 | 36.5 (25) | 34.5 (26) | -2.0 (-7.2, 3.2) | 23 (33.3) | 0.99 |
| Sleep disturbances | 20.5 (25) | 21.6 (29) | +1.1 (-2.0, 4.2) | 58 (21.1) | 21.6 (29) | 24.8 (29) | +3.2 (-3.9, 10.2) | 20 (27.0) | 0.15 | 27.5 (31) | 27.1 (30) | -0.5 (-8.1, 7.1) | 16 (22.5) | 0.99 |
| HADS (0-21) | | | | | | | | | | | | | | |
| Anxiety | 3.9 (3.4) | 3.9 (3.4) | 0.0 (-0.4, 0.3) | 54 (22.1) | 4.3 (3.3) | 4.3 (3.5) | -0.5 (-0.6, 0.5) | 12 (21.4) | 0.97 | 5.0 (4.3) | 4.9 (4.0) | -0.1 (-0.8, 0.6) | 18 (29.5) | 0.29 |
| Depression | 3.4 (3.1) | 3.8 (3.4) | 0.4 (0.0, 0.7) | 67 (27.5) | 4.1 (3.0) | 4.1 (3.5) | 0.0 (-0.7, 0.7) | 14 (25.0) | 0.73 | 4.4 (3.7) | 4.6 (3.9) | 0.2 (-0.6, 1.0) | 16 (26.2) | 0.81 |

[a] i.e. ≥10 point decrease in HRQoL functional scales or ≥10 point increase in symptoms scales, and ≥1.5 points increase in HADS scales. COVID-19 = Coronavirus disease 2019; CRC = Colorectal cancer; TC/VC = Telephone consultation/video consultation; HRQoL = Health-related quality of life; HADS = Hospital Anxiety and Depression Scale.

[b] P value for difference in prevalence of worsened HRQoL and HADS between groups, using logistic regression models (2-sided), adjusted for: age, sex, and number of comorbidities. Group numbers differ from **Table 1** and **Table 4** as these depend on the availability of pre-COVID questionnaires and current treatment status.

### Figure legends

**Figure 1.** Flowchart of COVID-19 study within the PLCRC cohort. [a] Total number of patients on April 17, 2020. [b] Patients who deceased or patients without informed consent for receiving questionnaires. [c] No matching pre-COVID PLCRC questionnaire available within one year pre-COVID, i.e. January 1, 2019 to January 31, 2020. [d] No data available on changes in clinical care planning. PLCRC = Prospective Dutch Colorectal Cancer cohort; COVID = Coronavirus disease.

**Figure 2.** Evaluation of TC and VC among CRC patients who had their face-to-face appointment changed into a TC or VC (n=379). CRC = Colorectal cancer; TC = Telephone consultations; VC = Video consultations.

Figure 1

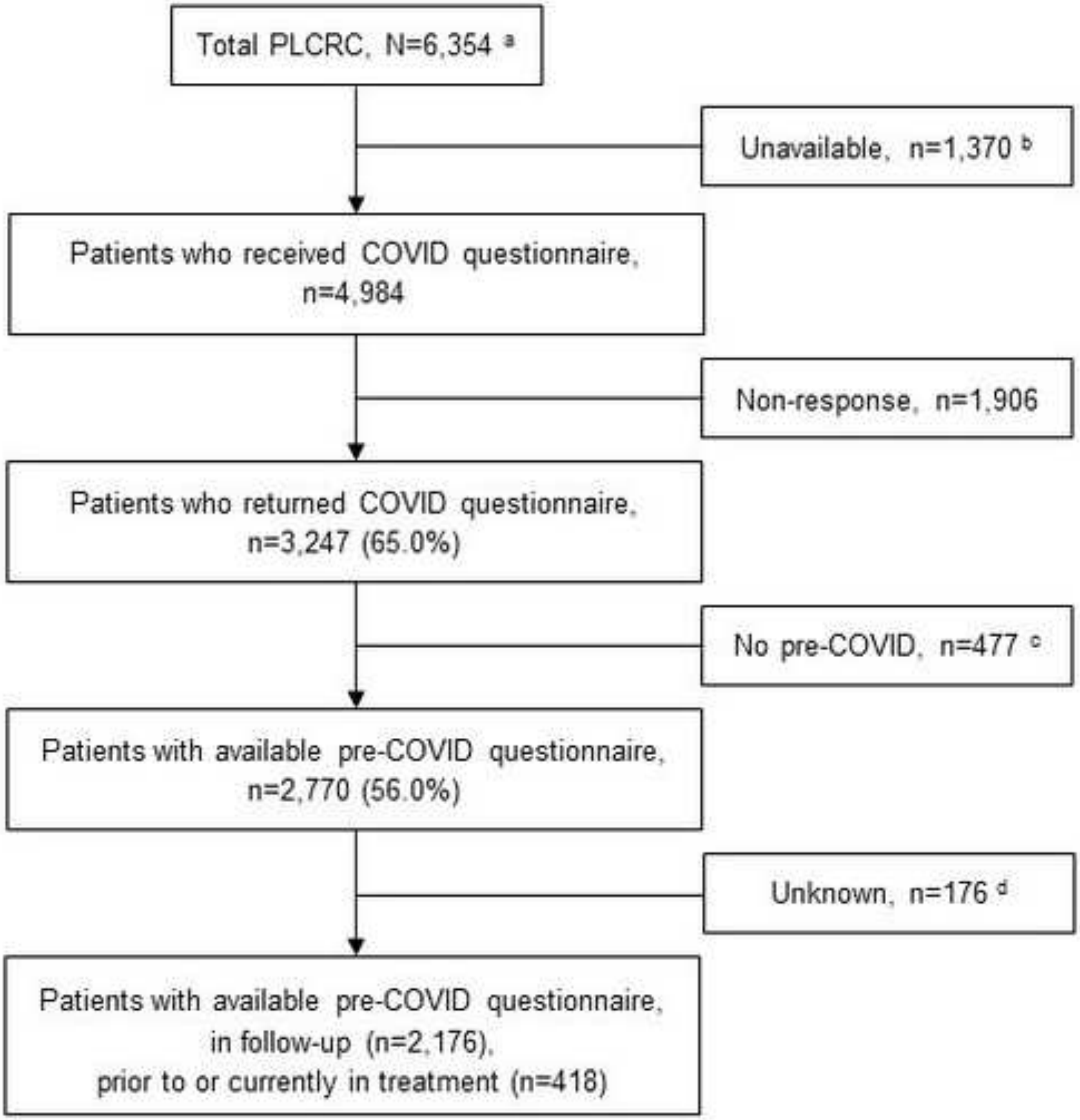

Total PLCRC, N=6,354 a
Unavailable, n=1,370 b
Patients who received COVID questionnaire, n=4,984
Non-response, n=1,906
Patients who returned COVID questionnaire, n=3,247 (65.0%)
No pre-COVID, n=477 c
Patients with available pre-COVID questionnaire, n=2,770 (56.0%)
Unknown, n=176 d
Patients with available pre-COVID questionnaire, in follow-up (n=2,176), prior to or currently in treatment (n=418)

Figure 2--FINAL

Figure 2

## Supplementary Material

pkab047_Supplementary_Data

