# Colorectal Cancer Care and Patients’ Perceptions Before and During COVID-19: Implications for Subsequent SARS-CoV-2 Infection Waves

**DOI:** 10.1093/jncics/pkab047

**Published:** 2021-05-13

**Authors:** Jeroen W G Derksen, Anne M May, Lonneke V van de Poll-Franse, Belle H de Rooij, Dorothee A Hafkenscheid, Helena M Verkooijen, Miriam Koopman, Geraldine R Vink

**Affiliations:** 1 Julius Center for Health Sciences and Primary Care, University Medical Center Utrecht, Utrecht University, Utrecht, the Netherlands; 2 Department of Medical Oncology, University Medical Center Utrecht, Utrecht University, Utrecht, the Netherlands; 3 Department of Research and Development, Netherlands Comprehensive Cancer Organisation (IKNL), Utrecht, the Netherlands; 4 Division of Psychosocial Research & Epidemiology, the Netherlands Cancer Institute, Amsterdam, the Netherlands; 5 Department of Medical and Clinical Psychology, Center of Research on Psychological and Somatic Disorders (CoRPS), Tilburg University, Tilburg, the Netherlands; 6 Imaging Division, University Medical Center Utrecht, Utrecht University, Utrecht, the Netherlands

## Abstract

**Background:**

Changes in colorectal cancer (CRC) care planning because of the coronavirus disease 2019 (COVID-19) pandemic and associated health-related quality of life (HRQoL) and well-being of patients with CRC are unknown. We report changes in CRC care and patient-reported outcomes including HRQoL, distress, and loneliness during the first wave of severe acute respiratory syndrome coronavirus 2 (SARS-CoV-2).

**Methods:**

In April 2020, 4984 patients included in the nationwide Prospective Dutch Colorectal Cancer cohort were invited to complete a COVID-19–specific questionnaire, together with the validated European Organisation for Research and Treatment of Cancer Core Quality of Life Questionnaire (QLQ-C30), De Jong Gierveld, and Hospital Anxiety and Depression Scale. Clinical data were obtained from the Netherlands Cancer Registry. Scores were compared with the year prior to COVID-19 and with an age- and sex-matched control population during COVID-19.

**Results:**

In total, 3247 (65.1%) patients responded between April and June 2020. Of the patients, 17% had canceled, postponed, or changed hospital visits to a telephone or video consult, and 5.3% had adjusted, postponed, or canceled treatment. Compared with controls, patients reported worse HRQoL but comparable distress and less social loneliness (patients = 21.2%; controls = 32.9%). Compared with pre–COVID-19, clinically meaningful deterioration of HRQoL was more prevalent in patients with changes in cancer care planning than in patients without changes. Prior to undergoing or currently undergoing treatment and infection worries were associated with lower HRQoL.

**Conclusions:**

CRC patients reported equal anxiety and depression but worse HRQoL than the control population. Changes in care planning were associated with deterioration of HRQoL and increased anxiety. In case of 1 or more risk factors, health-care specialists should discuss (mental) health status and possible support during future SARS-CoV-2 infection waves or comparable pandemics.

In March 2020, shortly after the outbreak of the coronavirus disease 2019 (COVID-19) pandemic, hospitals were suddenly required to reorganize to optimize clinical care for patients with COVID-19 and lower the risk of infection for all others involved. Consequently, ensuring the timely and optimal non–COVID-19 care has become increasingly challenging. Moreover, because of the absence of herd immunity and incomplete administration of all inhabitants with an effective COVID-19 vaccine, most countries will face multiple “waves” of infections. Current projections estimate that the COVID-19 pandemic could last years, possibly leading to prolonged or intermittent measures ranging from social distancing to (partial) lockdowns, ultimately to maintain intensive care capacity (1). Therefore, it is important to ensure that patients continue to receive essential care while minimizing their risk of severe acute respiratory syndrome coronavirus 2 (SARS-CoV-2) infection and to evaluate how initial clinical reorganizations were perceived by patients in terms of health-related quality of life (HRQoL) and mental well-being.

Here, we aim to: 1) evaluate changes in colorectal cancer (CRC) treatment and follow-up care during the first wave of SARS-CoV-2 infections in the Netherlands; 2) compare HRQoL and well-being of CRC patients with an age- and sex-matched noncancer control population; 3) study whether CRC patients experience clinically meaningful changes in HRQoL and mental well-being during the COVID-19 outbreak, as compared with the year before COVID-19; and 4) investigate which sociodemographic and clinical factors are related to changes in patient-reported outcomes (PROs).

## Methods

### Design and Setting

The current study is an observational study embedded in the ongoing nationwide Prospective Dutch Colorectal Cancer (PLCRC) cohort, an initiative coordinated by the Dutch Colorectal Cancer Group. PLCRC consists of patients diagnosed with a malignancy of the colon and/or rectum (*International Classification of Diseases*, 10th edition [ICD-10]: C18-20) in the Netherlands. The PLCRC study protocol, described in more detail elsewhere (2), has been approved by the Medical Research Ethics Committee Utrecht, the Netherlands. At PLCRC entry, participants provide informed consent and can opt to receive longitudinal questionnaires, which allows for a swift implementation of questionnaire-based observational studies. Clinical data was obtained through linkage with the Netherlands Cancer Registry.

### Study Population

In total, 4984 PLCRC participants who provided informed consent for receiving questionnaires were invited to participate in the current study and received an additional COVID-19–specific questionnaire on oncologic practice patterns, cancer care, HRQoL, and well-being during the COVID-19 outbreak. During the first national lockdown from March 23 to June 1, 2020, the COVID-19–specific questionnaire was sent out on April 17, 2020, and was received until June 1, 2020. Patients were included in the longitudinal analysis when a previous questionnaire of PLCRC was completed in the year before the COVID-19 pandemic (ie, between January 1, 2019, and January 31, 2020).

### Noncancer Control Population

Normative data on quality of life, symptoms, functioning, and comorbidities from a representative sample of the Dutch population was available from the Patient Reported Outcomes Following Initial Treatment and Long term Evaluation of Survivorship (PROFILES) registry and collected between May 4 and May 26, 2020 (3). This noncancer control population was matched to the PLCRC population based on a frequency distribution over age (younger than 30 years, 30-49, 50-69, 70 years and older) and sex strata to maximize the number of control participants matched to patients (ratio patients to norm 1:0.4).

### Data Collection

Sociodemographic and clinical data were obtained from the Netherlands Cancer Registry and include age, sex, date of primary cancer diagnosis, tumor location, stage at diagnosis, primary treatment received, and time since diagnosis (categorized). The ICD-10 was used to classify tumor types, and the TNM classification system was used to classify disease stage (4,5). Additional patient-reported sociodemographic data included educational level, marital status or partnership, living situation, height, and weight. Patient-reported clinical variables included current presence of metastases, current treatment status, and presence of comorbidities [adapted self-administered comorbidity questionnaire (6)]. Detailed information on data categorization and design of questionnaires can be found in the Supplementary Methods (available online).

***COVID-19–Specific Questionnaire (Only During COVID-19).*** Questions regarding SARS-CoV-2 infection status, the fear of getting COVID-19, and the impact on cancer treatment and follow-up care as experienced by the patient were developed by the Dutch Federation of Cancer Patients Organisations and the PROFILES Registry Group. Experiences with telephone or/or video consultation (TC/VC) were assessed based on questions developed by Barsom et al. (7), and the European Organisation for Research and Treatment of Cancer Core Quality of Life Questionnaire (EORTC QLQ-C30) response format was applied.

***HRQoL Questionnaire (Pre- and During COVID-19).*** The validated European Organisation for Research and Treatment of Cancer (EORTC) QLQ-C30 (8) was used to evaluate the patients’ quality of life, daily functioning, and complaints of common symptoms. Only during COVID-19, an additional single-item scale was added to assess worries about future health [from EORTC library (9)]. Linear transformation was used to obtain scores ranging from 0 to 100, where a higher score represents a higher (“better”) level of functioning or higher (“worse”) level of symptoms (10).

***HADS Questionnaire (Pre- and During COVID-19).*** Anxiety and depressive symptoms of patients were assessed using the Hospital Anxiety and Depression Scale (HADS) (11). To identify a clinically relevant level of anxiety or depression, a cutoff score of 8 was used (12).

***Loneliness Questionnaire (Only During COVID-19).*** Loneliness was assessed using the De Jong Gierveld short scales for social and emotional loneliness (13).

### Statistical Analyses

Patient characteristics were compared with the matched noncancer control population by means of the independent samples *t* test or χ^2^ test. To facilitate the interpretation of cross-sectional HRQoL measures, prevalences of patients and control participants exceeding thresholds for clinical importance (TCIs) were compared (14). General Linear Models and logistic and ordinal regression models were used to compare well-being of CRC patients with the matched control population, while adjusting for education level, marital status, living situation, comorbidities, and COVID-19 status. Mean changes in HRQoL, anxiety, and depression from less than 1 year pre–COVID-19 to during the COVID-19 pandemic were analyzed using the paired samples *t* test. In the subgroup of patients in treatment, a sensitivity analysis was performed by metastatic vs nonmetastatic disease. Logistic regression was used to test differences in prevalence of patients reporting a clinically meaningful worsening of HRQoL [ie, ≥10 points (15)], anxiety, and depression [ie, ≥1.5 points (16)] by alterations in cancer care planning, while adjusting for age, sex, and comorbidities. Here, the Holm’s sequential Bonferroni procedure was used to deal with inflated family-wise error rates because of multiplicity (17). Lastly, multivariable linear regression models including a priori selected variables were used to determine factors associated with changes in global quality of life, anxiety, and depression from pre–COVID-19 to during the COVID-19 outbreak. A *P* value of less than .05 was considered statistically significant unless otherwise stated, and all tests were 2-sided.

## Results

### Respondent Characteristics

**Figure 1.**
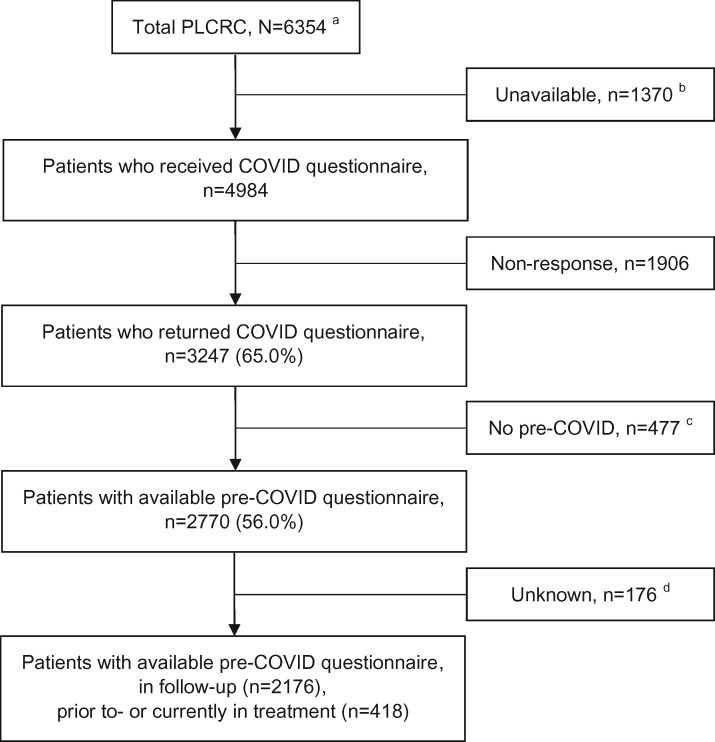
Flowchart of COVID-19 study within the PLCRC cohort. ^a^Total number of patients on April 17, 2020. ^b^Patients who deceased or patients without informed consent for receiving questionnaires. ^c^No matching pre-COVID PLCRC questionnaire available within 1 year pre-COVID (ie, January 1, 2019 to January 31, 2020). ^d^No data available on changes in clinical care planning. PLCRC = Prospective Dutch Colorectal Cancer cohort; COVID = coronavirus disease.

A total of 3247 patients (65.1%) returned their COVID-19–specific questionnaire (Figure 1). A total of 2664 noncancer control participants (75.9%) completed the COVID-19–specific questionnaire, of whom 1114 were age- and sex-matched to the patients.

Participants had a mean age of 67 (SD = 10) years, 63.1% were male, and 19.7% of the patients reported presence of metastases (Tables 1 and 2). Patients reported more comorbidities, were higher educated, more often married, and more often lived with a partner without children, compared with the control population. Changes in hospital visits or treatment plans were associated with presence of comorbidities (respectively, 37.0% and 41.5% with ≥2 comorbidities vs 33.4% in the total CRC patient population).

**Table 1.** Sociodemographic, comorbidity, and COVID-19 characteristics of CRC patients (n = 3247), subgroups according to changes in treatment or hospital visits and the age- and sex-matched noncancer control population (n = 1114)

Characteristics	Total CRC study population	Patients without changes in treatment or visits^c^	Patients with changes in hospital visits	Patients with changes in treatment	*P* ^d^	Matched control population	*P* ^e^
Total No.	3247	2429	551	171		1114	
Mean age (SD), y	67.1 (10.1)	67.3 (9.9)	66.1 (10.2)	66.9 (11.6)	.06	66.7 (12.0)	.31
≥70 years, No. (%)	1406 (43.3)	1057 (43.6)	218 (39.7)	77 (45.0)	.22	482 (43.3)	.98
Sex (men), No. (%)	2049 (63.1)	1547 (63.7)	342 (62.1)	107 (62.6)	.76	703 (63.1)	1.00
Education level, No. (%)^a^					.31		<.001
Low	913 (28.3)	702 (29.0)	142 (26.1)	40 (23.5)		458 (41.2)	
Medium	977 (30.3)	723 (29.9)	176 (32.3)	51 (30.0)		246 (22.1)	
High	1336 (41.4)	992 (41.0)	227 (41.7)	79 (46.5)		407 (36.6)	
Marital status (partner), No. (%)	2638 (81.7)	1982 (81.9)	441 (81.1)	137 (81.1)	.88	753 (67.6)	<.001
Living situation, No. (%)					.08		<.001
Alone	575 (17.8)	434 (17.9)	101 (18.5)	30 (17.5)		285 (25.6)	
Partner, no children	2187 (67.5)	1658 (68.3)	365 (66.8)	104 (60.8)		577 (51.8)	
Partner and children	267 (8.2)	193 (8.0)	40 (7.3)	23 (13.5)		176 (15.8)	
No partner, with children	140 (4.3)	99 (4.1)	22 (4.0)	10 (5.8)		28 (2.5)	
Other	69 (2.1)	43 (1.8)	18 (3.3)	4 (2.3)		48 (4.3)	
Mean (SD) BMI, kg/m^2^	26.5 (4.3)	26.5 (4.3)	26.4 (4.1)	26.5 (5.4)	.85	26.4 (4.4)	.41
BMI, No. (%)							
<25 kg/m^2^	1296 (40.2)	962 (39.8)	226 (41.4)	75 (44.4)	.66	467 (42.0)	.56
25 to <30 kg/m^2^	1366 (42.4)	1030 (42.7)	230 (42.1)	63 (37.3)		455 (41.0)	
≥30 kg/m^2^	561 (17.4)	423 (17.5)	90 (16.5)	31 (18.3)		189 (17.0)	
Comorbidities, No. (%)					.02		<.001
No	1184 (36.5)	914 (37.6)	177 (32.1)	54 (31.6)		345 (31.0)	
1	977 (30.1)	728 (30.0)	170 (30.9)	46 (26.9)		312 (28.0)	
≥2	1086 (33.4)	787 (32.4)	204 (37.0)	71 (41.5)		457 (41.0)	
SARS-CoV-2 virus infection, No. (%)					.17		.004
Yes, tested positive	9 (0.3)	5 (0.2)	1 (0.2)	2 (1.2)		2 (0.2)	
Probably, symptoms^b^ and/or fever	182 (5.7)	136 (5.6)	36 (6.5)	7 (4.1)		67 (6.0)	
No, tested negative	84 (2.6)	62 (2.6)	14 (2.5)	7 (4.1)		9 (0.8)	
No, not tested and no symptoms	2944 (91.5)	2217 (91.6)	499 (90.7)	153 (90.5)		1036 (93.0)	

a Low = secondary education (high school) or lower; medium = secondary (vocational) education; high = higher (vocational) education/university. BMI = body mass index; COVID-19 = coronavirus 19; CRC = colorectal cancer; SARS-CoV-2 = severe acute respiratory syndrome coronavirus 2.

b Respiratory symptoms and/or fever (38°C or higher) and/or 2 or more symptoms of flu or sickness, muscle pain, eye pain, or headache, and/or I have been in contact with someone who is (had been) infected with COVID-19. Missing: education level (n = 21 patients, n = 3 controls), living situation (n = 9 patients), BMI (n = 24 patients, n = 3 controls), SARS-CoV-2 infection (n = 28 patients).

c Missing: data on changes in care planning (n = 96 patients). Percentages do not always add up to 100 because of rounding to whole numbers.

d *P* values for comparisons by changes in treatment or hospital visits using 1-way analysis of variance and χ^2^ test.

e *P* values for comparison between total CRC study population and the matched control population using independent samples *t* test and χ^2^ test.

**Table 2.** Disease and treatment characteristics of CRC patients (n = 3247)

Characteristics	Total CRC study population	Patients without changes in treatment or visits^c^	Patients with changes in hospital visits	Patients with changes in treatment	*P* ^d^
Total No. (%)	3247	2429 (75)	551 (17)	171 (5)	
Tumor type, No. (%)					.45
Colon, C18.0-18.9	1448 (44.6)	1092 (45.0)	232 (42.1)	79 (46.2)	
Rectum, C19.9, C20.9	1047 (32.2)	793 (32.6)	176 (31.9)	52 (30.4)	
Unknown^a^	752 (23.2)	544 (22.4)	143 (26.0)	40 (23.4)	
Tumor stage at diagnosis, No. (%)					
Stage I	516 (15.9)	397 (16.3)	85 (15.4)	16 (9.4)	<.001
Stage II	583 (18.0)	449 (18.5)	99 (18.0)	17 (9.9)	
Stage III	1101 (33.9)	833 (34.3)	178 (32.3)	65 (38.0)	
Stage IV	286 (8.8)	199 (8.2)	44 (8.0)	33 (19.3)	
Not applicable	9 (0.3)	7 (0.3)	2 (0.4)	0 (0)	
Unknown^a^	752 (23.2)	544 (22.4)	143 (26.0)	40 (23.4)	
Time since primary diagnosis, No. (%)					.12
<6 mo	313 (9.6)	225 (9.3)	62 (11.3)	14 (8.2)	
6 to <12 mo	568 (17.5)	407 (16.8)	108 (19.6)	34 (19.9)	
12 to <24 mo	955 (29.4)	702 (28.9)	175 (31.8)	52 (30.4)	
24 to <60 mo	1085 (33.4)	844 (34.7)	159 (28.9)	49 (28.7)	
≥60 mo	250 (7.7)	195 (8.0)	35 (6.4)	16 (9.4)	
Unknown^a^	76 (2.3)	56 (2.3)	12 (2.2)	6 (3.5)	
Current presence of metastases, No. (%)^b^					<.001
Yes	640 (19.7)	438 (19.5)	102 (19.0)	83 (49.4)	
No	2119 (65.3)	1627 (72.5)	365 (68.1)	66 (39.3)	
Don’t know	271 (8.3)	179 (8.0)	69 (12.9)	19 (11.3)	
Current phase of disease/treatment, No. (%)^b^					<.001
Still have to start Tx	27 (0.8)	15 (0.6)	6 (1.1)	6 (3.5)	
Currently being treated	567 (17.5)	380 (15.6)	93 (16.9)	79 (46.2)	
Completed Tx, currently in follow-up care	2456 (75.6)	1859 (76.5)	445 (80.8)	84 (49.1)	
Completed Tx, no follow-up care	181 (5.6)	166 (6.8)	5 (0.9)	2 (1.2)	
Unknown	16 (0.5)	9 (0.4)	2 (0.4)	0 (0)	
Current treatment, or soon to be started, No. (%)^b^					
Chemotherapy	371 (11.4)	240 (10.4)	63 (11.5)	61 (35.7)	<.001
Surgery	141 (4.3)	104 (4.5)	24 (4.4)	9 (5.3)	.89
Immunotherapy	81 (2.5)	52 (2.3)	15 (2.7)	12 (7.0)	.001
Radiotherapy	48 (1.5)	38 (1.6)	4 (0.7)	5 (2.9)	.10
Targeted therapy	27 (0.8)	19 (0.8)	1 (0.2)	6 (3.5)	<.001
Symptom management	66 (2.0)	33 (1.4)	20 (3.7)	10 (5.8)	<.001
Active surveillance/wait and see	268 (8.3)	189 (8.2)	44 (8.1)	28 (16.4)	.001
Other	328 (10.1)	217 (9.4)	60 (11.0)	37 (21.6)	<.001
Current supportive care, No. (%)^b^					
Oncology nurse	509 (15.7)	361 (14.9)	97 (17.6)	39 (22.8)	.01
General practitioner	258 (7.9)	172 (7.1)	55 (10.0)	24 (14.0)	.001
Physical therapy	177 (5.5)	114 (4.7)	38 (6.9)	22 (12.9)	<.001
Psychological care	122 (3.8)	68 (2.8)	33 (6.0)	16 (9.4)	<.001
Dietician	103 (3.2)	76 (3.1)	21 (3.8)	6 (3.5)	.71
Oncological rehabilitation	67 (2.1)	37 (1.5)	16 (2.9)	9 (5.3)	.001
Other	391 (12.0)	249 (10.3)	92 (16.7)	32 (18.7)	<.001

a Unknown because clinical data was not yet available because of the time-lag within the Netherlands Cancer Registry. Percentages do not always add up to 100 because of rounding to whole numbers. COVID-19 = coronavirus 2019; CRC = colorectal cancer; Tx = treatment.

b Patient-reported data from COVID-19-specific questionnaire. Other supportive care: patient support groups, religious and/or spiritual care, sexologist, and creative therapy.

c Missing: data on changes in care planning (n = 96 patients).

d *P* values for comparisons by changes in treatment or hospital visits using χ^2^ test.

### Changes in Treatment and Follow-up Care During COVID-19

In our total study population, 17% of patients had canceled and/or postponed hospital visits or visits that were changed into a TV or VC, and 5.3% reported adjusted, postponed, and/or canceled treatment.

Of 594 patients prior to or currently in treatment during COVID-19, 86 patients (14.5%) reported postponed and/or canceled hospital visits, and 128 patients (21.5%) had their visit changed to a TC or VC (Supplementary Table 1, available online). In 34 patients (5.7%), treatment was adjusted, in 48 patients (8.1%) postponed, and in 4 patients (0.7%) systemic or targeted treatment was canceled. In case of physical complaints or concerns, patients reported a barrier to contact their general practitioner (11.3%) or medical specialist and/or nurse (6.4%). These undesired hesitations were even more common in the noncancer control population (respectively, 37.9% and 31.3%).

Of 2456 patients with completed treatment, 363 patients (14.8%) had a hospital follow-up visit postponed and/or canceled, and 259 patients (10.5%) had a visit changed to a TC or VC (Supplementary Table 1, available online). In case of physical complaints or concerns, these patients reported they contacted their general practitioner (18.9%) or medical specialist or nurse (14.0%) less quickly as compared with pre–COVID-19, and, respectively, 70.8% and 60.2% reported no difference as compared with pre–COVID-19.

**Figure 2.**
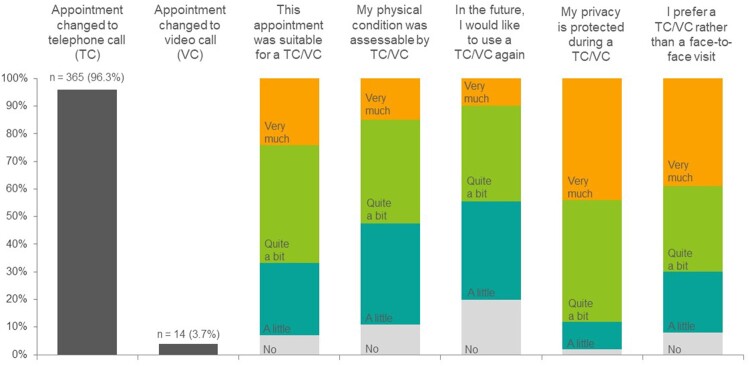
Evaluation of TC and VC among CRC patients who had their face-to-face appointment changed into a TC or VC (n = 379). CRC = colorectal cancer; TC = telephone consultations; VC = video consultations.

For 379 patients (12.2%), a face-to-face visit was changed to a TC (n = 365) or VC (n = 14). Most patients reported this change to be very or quite suitable (66.6%), but 47.1% reported that their physical condition could not or poorly be assessed (Figure 2). The majority (87.8%) was not worried about privacy issues, and although 44.9% would use a TC or VC again, a face-to-face consultation remained most preferred (70.4%), also in the subgroup of patients prior to or in treatment (80.7%).

### HRQoL and Mental Well-Being in CRC Patients vs Controls During COVID-19

Although on average, CRC patients did not exceed TCIs regarding HRQoL during the COVID-19 pandemic, a statistically significantly larger proportion of CRC patients reported levels of fatigue, sleep disturbance, and functional impairments that exceed TCIs, as compared with the noncancer control population (Table 3). Elevated levels of anxiety and depression were equally present in CRC patients and controls (anxiety: 11.3% vs 10.2%; depression: 11.4% vs 12.3%, respectively), but patients reported statistically significantly less moderate–severe loneliness—especially in terms of social loneliness (respectively, 21.2% vs 32.9%). Regarding concerns about getting SARS-CoV-2 infection, CRC patients were equally concerned as control participants.

**Table 3.** Quality of life (including daily functioning, fatigue, and sleep disturbances), anxiety, depression, and loneliness as reported by patients with CRC (n = 3247), compared with the matched noncancer control population during the COVID-19 pandemic (n = 1114).

Patient-reported outcome	Colorectal cancer patients^b^	Matched control population	*P* ^c^
Total No.	3247	1114	
HRQoL—EORTC QLQ-C30, 0-100, mean (SD)			
Global quality of life	76.7 (18)	74.2 (17)	.04
Physical functioning	86.4 (17)	89.6 (15)	<.001
Role functioning	82.5 (25)	88.8 (21)	<.001
Emotional functioning	86.5 (17)	87.9 (16)	<.001
Cognitive functioning	88.3 (17)	92.6 (14)	<.001
Social functioning	88.2 (20)	95.0 (14)	<.001
Fatigue	20.3 (22)	14.7 (19)	<.001
Sleep disturbances	17.6 (25)	14.8 (24)	<.001
Future health worries	27.2 (25)	21.6 (22)	<.001
Prevalence exceeding TCI, No. (%)^a^			
Physical functioning, <83	963 (29.8)	245 (22.0)	<.001
Role functioning, <58	459 (14.2)	96 (8.6)	<.001
Emotional functioning, <71	534 (16.5)	158 (14.2)	.006
Cognitive functioning, <75	609 (18.9)	115 (10.3)	<.001
Social functioning, <58	286 (8.9)	36 (3.2)	<.001
Fatigue, >39	503 (15.6)	106 (9.5)	<.001
Sleep disturbances, >50	361 (11.2)	102 (9.2)	.002
HADS, 0-21, mean (SD)			
Anxiety, 0-21	3.4 (3)	3.2 (3)	.05
Depression, 0-21	3.1 (3)	3.2 (3)	.15
Categorical, No. (%)			
Mild/severe anxiety, 8-21	365 (11.3)	114 (10.2)	.03
Mild/severe depression, 8-21	367 (11.4)	137 (12.3)	.24
Overall loneliness (0-6), mean (SD)	1.6 (2)	1.9 (2)	<.001
Categorical, No. (%)			.06
No loneliness, 0-1	1831 (57.0)	549 (49.3)	
Moderate loneliness, 2-4	1142 (35.6)	444 (39.9)	
Severe loneliness, 5-6	238 (7.4)	121 (10.9)	
Emotional loneliness, 0-3, mean (SD)	0.9 (1)	0.9 (1)	.45
Categorical, No. (%)			.001
No emotional loneliness, 0-1	2314 (72.4)	819 (73.5)	
Emotionally lonely, 2-3	880 (27.6)	295 (26.5)	
Social loneliness, 0-3, mean (SD)	0.7 (1.1)	1.0 (1.2)	<.001
Categorical, No. (%)			<.001
No social loneliness, 0-1	2514 (78.8)	748 (67.1)	
Socially lonely, 2-3	677 (21.2)	366 (32.9)	
Worried about getting SARS-CoV-2 infection, No. (%)			.03
Not at all	590 (18.4)	198 (17.8)	
A little	1935 (60.2)	724 (65.1)	
Quite a bit	573 (17.8)	150 (13.5)	
Very much	117 (3.6)	40 (3.6)	

a TCIs for the functioning and symptom scales of the EORTC QLQ-C30 (ie, below the TCI for functioning scales or above the TCI for symptom scales indicate clinically important problems or symptoms). No TCI available for global quality of life and future health worries. COVID-19 = coronavirus 2019; CRC = colorectal cancer; EORTC QLQ-C30 = European Organisation for Research and Treatment of Cancer Quality of Life Questionnaire C30; HADS = Hospital Anxiety and Depression Scale; HRQoL = health-related quality of life; SARS-CoV-2 = severe acute respiratory syndrome coronavirus 2; TCI = thresholds for clinical importance.

b Missing: QLQ-C30 (n = 15-45, 0.5-1.4%), HADS (n = 17, 0.4%), loneliness (n = 36, 0.8%), emotional loneliness (n = 53, 1.2%), social loneliness (n = 56, 1.3%), worried about SARS-CoV-2 infection (n = 34, 0.8%). Percentages do not always add up to 100 because of rounding to whole numbers.

c *P* values for comparison between colorectal cancer patients and the matched control population using General Linear Models, ordinal and logistic regression models (2-sided), adjusted for education level, marital status, living situation, comorbidities, and COVID-19 status.

### Changes in HRQoL and Mental Well-Being From Pre–COVID-19 to During COVID-19

Patients in follow-up (Table 4) who reported to have had changed, postponed or canceled hospital visits, compared with patients without changes in cancer care planning, more frequently reported a clinically meaningful decrease in role (respectively, 20.8% vs 16.4%), emotional (respectively, 18.7% vs 10.3%), and social functioning (respectively, 15.1% vs 11.1%) and more often an increase in fatigue (respectively, 27.2% vs 18.1%), sleep disturbance (respectively, 17.7% vs 12.1%), and anxiety (respectively, 25.1% vs 18.9%).

**Table 4.** Changes in quality of life, anxiety, and depression from less than 1 year pre–COVID-19 to during the COVID-19 pandemic in patients with CRC who completed treatment and were in follow-up, stratified for changes in cancer care planning

Patient-reported outcome	CRC patients without changes in treatment plan or hospital visits (n = 1786)				CRC patients with hospital visits canceled, postponed, or changed into TC or VC (n = 390)				
	Pre-COVID, mean (SD)	During COVID, mean (SD)	Within group change (95% CI)	Clinically meaningful worsening^a^ No. (%)	Pre-COVID, mean (SD)	During COVID, mean (SD)	Within group change (95% CI)	Clinically meaningful worsening No. (%)^a^	*P* ^b^
HRQoL (0-100)									
Global quality of life	80.8 (16)	80.4 (16)	-0.4 (-1.1 to -0.3)	312 (17.6)	75.4 (18)	74.9 (17)	-0.5 (-2.1 to 1.0)	74 (19.0)	.49
Physical functioning	88.7 (15)	88.8 (15)	+0.2 (-0.3 to 0.7)	174 (9.8)	86.8 (16)	86.9 (16)	+0.1 (-1.2 to 1.4)	49 (12.6)	.19
Role functioning	84.8 (23)	87.5 (21)	+2.7 (1.7 to 3.8)	293 (16.4)	78.8 (26)	81.8 (24)	+3.0 (0.5 to 5.5)	81 (20.8)	.03
Emotional functioning	87.7 (17)	89.0 (15)	+1.3 (0.7 to 2.0)	183 (10.3)	83.4 (19)	83.0 (19)	-0.3 (-2.1 to 1.6)	73 (18.7)	<.00^c^
Cognitive functioning	88.2 (16)	90.2 (16)	+2.0 (1.3 to 2.6)	254 (14.2)	84.3 (19)	86.5 (18)	+2.2 (0.6 to 3.8)	61 (15.6)	.61
Social functioning	88.8 (18)	92.5 (18)	+3.7 (2.8 to 4.5)	198 (11.1)	82.9 (22)	87.6 (20)	+4.7 (2.5 to 7.0)	59 (15.1)	.03
Fatigue	19.9 (21)	15.5 (19)	-4.4 (-5.3 to -3.6)	323 (18.1)	26.2 (23)	22.8 (24)	-3.3 (-5.5 to -1.2)	106 (27.2)	<.001^c^
Sleep disturbances	17.5 (24)	14.4 (22)	-3.1 (-4.1 to -2.1)	215 (12.1)	22.5 (27)	20.2 (27)	-2.2 (-5.0 to 0.6)	69 (17.7)	.01
HADS (0-21)									
Anxiety	3.1 (3.1)	3.0 (3.1)	-0.1 (-0.2 to 0.0)	289 (18.9)	3.9 (3.6)	4.0 (3.4)	0.2 (-0.1 to 0.5)	85 (25.1)	.01
Depression	2.8 (3.1)	2.6 (3.0)	-0.1 (-0.3 to 0.0)	247 (16.2)	3.8 (3.7)	3.6 (3.5)	-0.2 (-0.5 to 0.1)	54 (16.0)	.92

a That is, ≥10 point decrease in HRQoL functional scales or ≥10 point increase in symptoms scales and ≥1.5 points increase in HADS scales. COVID-19 = coronavirus disease 2019; CRC = colorectal cancer; HADS = Hospital Anxiety and Depression Scale; HRQoL = health-related quality of life; TC or VC = telephone consultation or video consultation.

b *P* values for difference in prevalence of worsened HRQoL and HADS between groups, using logistic regression models (2-sided), adjusted for age, sex, and number of comorbidities. Group numbers differ from Table 1 as these depend on the availability of pre-COVID questionnaires.

c Statistically significant after Holm’s Sequential Bonferroni correction.

In patients in treatment (Table 5), clinically meaningful worsening of HRQoL, anxiety, and depression was more prevalent compared with patients in follow-up. In those with adjusted/postponed/canceled treatment, compared with patients without changes in cancer care planning, clinically meaningful deterioration occurred most for physical (respectively, 29.0% vs 23.4%), cognitive (respectively, 30.4% vs 23.7%), and social functioning (respectively, 34.8% vs 28.8%), as well as for anxiety (respectively, 29.5% vs 22.1%). Sensitivity analysis within this group showed no statistically significant differences in HRQoL, depression, and anxiety between patients with metastatic vs nonmetastatic disease in neither of the 3 settings of cancer care planning (data not shown).

**Table 5.** Changes in quality of life, anxiety, and depression from less than 1 year pre–COVID-19 to during the COVID-19 pandemic in patients with CRC who were prior to or currently in treatment, stratified for changes in cancer care planning

Patient-reported outcome	CRC patients without changes in treatment plan or hospital visits (n = 275)				CRC patients with hospital visits canceled, postponed, or changed into TC or VC (n = 74)					CRC patients with adjusted, postponed, or canceled treatment (n = 69)				
	Pre-COVID, mean (SD)	During COVID, mean (SD)	Within group change (95% CI)	Clinically meaningful worsening^a^ No. (%)	Pre-COVID, mean (SD)	During COVID, mean (SD)	Within group change (95% CI)	Clinically meaningful worsening^a^ No. (%)	*P* ^b^	Pre-COVID, mean (SD)	During COVID, mean (SD)	Within group change (95%CI)	Clinically meaningful worsening^a^ No. (%)	*P* ^b^
HRQoL (0-100)														
Global quality of life	71.5 (19)	66.9 (20)	-4.6 (-7.4 to -1.9)	95 (34.9)	69.4 (17)	66.4 (21)	-3.0 (-7.1 to 1.1)	19 (26.4)	.25	71.4 (22)	66.4 (22)	-5.0 (-9.8 to -0.1)	21 (30.4)	.48
Physical functioning	84.8 (16)	81.4 (18)	-3.4 (-5.3 to -1.4)	64 (23.4)	81.4 (17)	78.9 (19)	-2.5 (-5.4 to 0.4)	16 (21.6)	.96	81.4 (21)	78.6 (20)	-2.7 (-6.5 to 1.1)	20 (29.0)	.34
Role functioning	72.4 (29)	71.1 (29)	-1.3 (-5.1 to 2.6)	85 (30.9)	70.9 (29)	70.7 (31)	-0.2 (-7.2 to 6.8)	21 (28.4)	.97	68.8 (34)	67.4 (32)	-1.4 (-8.7 to 5.8)	24 (34.8)	.68
Emotional functioning	81.4 (18)	81.8 (19)	+0.5 (-1.6 to 2.5)	40 (14.6)	81.5 (19)	82.1 (19)	+0.6 (-3.3 to 4.5)	13 (17.8)	.35	72.3 (22)	74.9 (22)	+2.5 (-2.3 to 7.4)	11 (15.9)	.86
Cognitive functioning	86.0 (17)	84.9 (19)	-1.1 (-3.2 to 1.0)	65 (23.7)	83.3 (17)	85.2 (16)	+1.8 (-2.4 to 6.0)	17 (23.3)	.91	81.4 (21)	80.9 (19)	-0.5 (-4.7 to 3.7)	21 (30.4)	.33
Social functioning	79.1 (23)	78.6 (23)	-0.5 (-3.5 to 2.5)	79 (28.8)	80.1 (25)	76.9 (25)	-3.2 (-8.9 to 2.5)	23 (31.5)	.66	77.5 (25)	74.6 (28)	-2.9 (-9.1 to 3.3)	24 (34.8)	.46
Fatigue	30.1 (23)	30.0 (23)	-0.1 (-3.1 to 3.0)	89 (32.5)	30.5 (23)	31.2 (24)	+0.7 (-4.9 to 6.2)	26 (35.1)	.55	36.5 (25)	34.5 (26)	-2.0 (-7.2 to 3.2)	23 (33.3)	.99
Sleep disturbances	20.5 (25)	21.6 (29)	+1.1 (-2.0 to 4.2)	58 (21.1)	21.6 (29)	24.8 (29)	+3.2 (-3.9 to 10.2)	20 (27.0)	.15	27.5 (31)	27.1 (30)	-0.5 (-8.1 to 7.1)	16 (22.5)	.99
HADS (0-21)														
Anxiety	3.9 (3.4)	3.9 (3.4)	0.0 (-0.4 to 0.3)	54 (22.1)	4.3 (3.3)	4.3 (3.5)	-0.5 (-0.6 to 0.5)	12 (21.4)	.97	5.0 (4.3)	4.9 (4.0)	−0.1 (−0.8 to 0.6)	18 (29.5)	.29
Depression	3.4 (3.1)	3.8 (3.4)	0.4 (0.0 to 0.7)	67 (27.5)	4.1 (3.0)	4.1 (3.5)	0.0 (-0.7 to 0.7)	14 (25.0)	.73	4.4 (3.7)	4.6 (3.9)	0.2 (−0.6 to 1.0)	16 (26.2)	.81

a That is, ≥10 point decrease in HRQoL functional scales or ≥10 point increase in symptoms scales and ≥1.5 points increase in HADS scales. COVID-19 = coronavirus disease 2019; CRC = colorectal cancer; HADS = Hospital Anxiety and Depression Scale; HRQoL = health-related quality of life; TC or VC = telephone consultation or video consultation.

b *P* value for difference in prevalence of worsened HRQoL and HADS between groups, using logistic regression models (2-sided), adjusted for age, sex, and number of comorbidities. Group numbers differ from Tables 1 and 4 as these depend on the availability of pre-COVID questionnaires and current treatment status.

### Factors Related to Changes in HRQoL, Anxiety, and Depression From Pre– COVID-19 to During COVID-19

Being prior to or in treatment during the COVID-19 pandemic and being quite a bit or very worried about getting a SARS-CoV-2 infection were statistically significantly associated with a decrease in HRQoL (Supplementary Table 2, available online). Factors statistically significantly associated with an increase in anxiety levels were increasing age (0.01, 95% CI = 0.00 to 0.03; *P* = .03) and being quite a bit or very worried about a SARS-CoV-2 infection (1.0, 95% CI = 0.7 to 1.3; *P* < .001), and having a partner but no children (-0.6, 95% CI = -1.1 to -0.1; *P* = .02) was statistically significantly associated with lower anxiety levels. Factors associated with an increase in depression levels included increasing age (0.02, 95% CI = 0.01 to 0.03; *P* = .007), being female (0.2, 95% CI = 0.0 to 0.4; *P* = .04), being prior to or in treatment during the COVID-19 pandemic (0.6, 95% CI = 0.3 to 1.0; *P* = .001), and being quite a bit or very worried about a SARS-CoV-2 infection (0.7, 95% CI = 0.5 to 0.9; *P* < .001), and having metastatic disease (-0.4, 95% CI = -0.8 to -0.1; *P* = .001) was statistically significantly associated with lower depression levels.

## Discussion

During the first wave of SARS-CoV-2 infections in the Netherlands, hospital visits for patients with CRC were postponed or cancelled in 14.5% of patients in treatment and in 14.8% of patients in follow-up. Hospital visits were changed into a TC or VC for 21.5% of patients in treatment and in 10.5% of patients in follow-up. Although most patients reported this change to be very much or quite suitable, the vast majority favors a face-to-face consultation in the future. This pandemic has catalyzed the implementation of telemedicine to deliver health care, but remaining challenges for patients and medical staff require continued research and development to ultimately find implementation in routine health care (18,19). Moreover, anticancer treatments were adjusted in 5.7%, postponed in 8.1%, and canceled in 0.7% of the patients in treatment. During this early phase of the COVID-19 outbreak, a statistically significantly larger proportion of CRC patients reported levels of fatigue, sleep disturbance, and health-related functional impairments exceeding TCIs, compared with noncancer controls. Mild or severe anxiety and depression were reported equally, and patients reported statistically significantly less social loneliness.

In patients in follow-up during the COVID-19 pandemic who had hospital visits canceled, postponed, or changed into TC or VC, the proportion of clinically meaningful worsening of role, emotional and social functioning, fatigue, sleep disturbance, and anxiety was statistically significantly higher compared with patients without changes in cancer care planning. Interestingly, in patients who were prior to or in treatment during the COVID-19 pandemic, the differences between patients with and without changes in treatment were non-statistically significant. Nevertheless, in this group, the overall prevalence of clinically meaningfully deteriorated HRQoL and anxiety was higher compared with those in follow-up. Despite the absence of statistical significance, patients whose treatment was adjusted, postponed, or canceled reported more often a clinically meaningful increase in anxiety than patients without changes.

Other studies that investigated anxiety and depression in cancer patients during the COVID-19 outbreak reported mild–severe anxiety levels in 36%-56% of patients and mild–severe depression levels in 19%-47% of patients (20-23). This difference with our population (11.3%) might be explained by differences in study population, cancer types, and nationality. In a Dutch study in breast cancer patients, comparable mean levels of anxiety and depression were observed that also did not statistically significantly change from pre–COVID-19 to during the COVID-19 pandemic (24). Our results confirm previously reported data of 2 large Dutch cohort studies that reported on practice patterns and perceived HRQoL in patients with cancer during the COVID-19 outbreak (24,25). First, Bargon et al. (24) previously reported statistically significant but minimal changes in HRQoL scores from before to during COVID-19 in breast cancer patients and survivors. Second, van de Poll-Franse et al. (25), who included part of the current PLCRC data, reported no clinically meaningful differences in HRQoL scales between patients with various tumor types and a matched control population. The current analysis adds to these findings by showing that despite small average changes in HRQoL, a large proportion of CRC patients does experience health-related functional impairments and fatigue and sleep disturbance beyond thresholds for TCI—statistically significantly more than the age- and sex-matched noncancer control population. Additionally, we showed that changes in cancer care planning are associated with an increased prevalence of clinically meaningful deterioration of daily functioning, fatigue, sleep disturbance, and anxiety that are, as such, perceptible to patients.

A positive finding was that, compared with the control population, CRC patients experienced statistically significantly less social loneliness. Possibly, the government’s measures such as restricted freedom of movement and social distancing have less impact on cancer patients as they may already be much more cautious about infections. To some, the COVID-19 crisis may have distracted from focusing on their disease, which, together with existing or increased social support from friends and family, could have lessened the impact on psychosocial well-being.

Altogether, we emphasize that despite minimal average changes in HRQoL, anxiety, and depression from pre–COVID to during the COVID-19 pandemic, substantial differences do occur on an individual patient level. Therefore, individual- and cancer-specific factors related to changes in HRQoL and mental well-being were identified. Age, sex, having a partner but no children, metastatic disease, being in treatment, and being quite a bit or very much worried about getting a SARS-CoV-2 infection were factors statistically significantly associated with changes in global quality of life, anxiety, or depression. Although individual factors are only associated with small changes, they might be intertwined and could cumulatively lead to meaningful changes. These factors should be taken into consideration by health-care professionals and used to identify patients at risk of deterioration of HRQoL and mental well-being during subsequent waves of SARS-CoV-2 infections or comparable future pandemics. Especially in the group of patients presenting with 1 or more of the above-mentioned factors, discussing (mental) health status and possible support is recommended.

A limitation of the current study was, first, that some clinical information was self-reported by patients, which could have led to misclassification and therefore dilution of our results. Although expected to be of little influence (26), results may be prone to seasonality, as questionnaires were completed during spring 2020 and compared with 2019 year round. In contrast, PROs are highly valuable as a direct measure of how patients’ health and well-being were perceived. Second, the small number of patients in treatment with changes in cancer care might have influenced the magnitude of statistical significance. Therefore, it is important to consider the direction and size of the observed effect sizes. Third, because we lack information on reasons to not participate in this study, an under- or overestimation of the results because of selective response could not be ruled out. Lastly, we observed baseline differences between CRC patients and the control population, which could influence comparisons between the 2 groups, however, models were adjusted for the imbalanced factors. There are also several strengths related to this analysis, including the large number of respondents, which allowed for comprehensive analyses in a specific patient population. Moreover, the benefit of implementing this questionnaire-based study in an ongoing cohort with clinical data and PROs from diagnosis onward is that data before the COVID-19 outbreak were available allowing for a comparison with the year before. Additionally, this enables longitudinal modeling of repeated PROs during future SARS-CoV-2 infection waves and subsequent government-imposed national prevention measures. This will provide further insight in the impact of ongoing social distancing and other nationwide prevention measures on patients’ well-being and will help tailor mental support strategies.

To conclude, between 9.8% and 18.9% of patients without changes in cancer care during follow-up reported clinically meaningful worsening of HRQoL, anxiety, and depression compared with the year before the COVID-19 outbreak, and in patients prior to or in treatment, these prevalences increased to 14.6%-34.9%. Both in patients in treatment and in follow-up, changes in cancer care planning were associated with increased deterioration of several aspects of HRQoL and anxiety. Interestingly, during COVID-19, patients experienced statistically significantly less social loneliness than the noncancer control population. To optimally meet patient needs, (mental) health status and possible support options should be discussed—especially in patients presenting with 1 or more risk factors—during future SARS-CoV-2 infection waves or comparable pandemics.

## Funding

The current work was not supported by a specific funding source.

## Notes

**Role of the funder:** Not applicable.

**Disclosures:** MK reports institutional financial interests with Amgen, Bayer, BMS, Merck-Serono, Nordic Pharma, Roche, Servier, Sirtex, and Sanofi-Aventis. MK reports the following non-financial interests: an advisory role for ZonMw, membership of the scientific board of the Dutch Cancer Society (KWF), chairmanship of the Dutch Colorectal Cancer Group (DCCG), principal investigator (PI) of the Prospective Dutch Colorectal Cancer (PLCRC) cohort. GRV reports institutional financial interests with Servier, Merck, Bayer, Sirtex, BMS, and Lilly. All other authors report no competing interests.

**Acknowledgements:** Full list of consortium members and their affiliations for the PLCRC study group: Geerard L. Beets (Department of Surgery, Nederlands Kanker Instituut—Antoni van Leeuwenhoek Hospital, Amsterdam, the Netherlands); Eric J.Th. Belt (Department of Surgery, Albert Schweitzer Hospital, Dordrecht, the Netherlands); Maaike Berbée (Department of Radiotherapy, Maastro Clinic, Maastricht, the Netherlands); Frederique H. Beverdam (Department of Surgery, Franciscus Gasthuis & Vlietland Hospital, Schiedam, the Netherlands); Ruud Blankenburgh (Department of Medical Oncology, Saxenburgh Hospital, Hardenberg, the Netherlands); Peter Paul L.O. Coene (Department of Surgery, Maasstad Hospital, Rotterdam, the Netherlands); Hester van Cruijsen (Department of Medical Oncology, Antonius Hospital, Sneek, the Netherlands); Jan Willem T. Dekker (Department of Surgery, Reinier de Graaf Hospital, Delft, the Netherlands); Joyce M. van Dodewaard-de Jong (Department of Medical Oncology, Meander Medical Center, Amersfoort, the Netherlands); Frans L.G. Erdkamp (Department of Medical Oncology, Zuyderland Hospital, Heerlen, the Netherlands); Jan Willem B. de Groot (Department of Medical Oncology, Isala Oncology Center, Zwolle, the Netherlands); Annebeth W. Haringhuizen (Department of Medical Oncology, Ziekenhuis Gelderse Vallei, Ede, the Netherlands); Helgi H. Helgason (Department of Medical Oncology, Haaglanden Medical Center, Den Haag, the Netherlands); Mathijs P. Hendriks (Department of Medical Oncology, Northwest Clinics, Alkmaar, the Netherlands); Ignace H.J.T. de Hingh (Department of Surgery, Catharina Hospital, Eindhoven, the Netherlands); Ronald Hoekstra (Department of Medical Oncology, Ziekenhuisgroep Twente, Hengelo, the Netherlands); Jan N.M. Ijzermans (Department of Surgery, Erasmus MC, University Medical Center Rotterdam, Rotterdam, the Netherlands); Jan Jansen (Department of Surgery, Admiraal de Ruyter Hospital, Goes, the Netherlands); Frank W.H. Kloppenberg (Department of Surgery, Treant Hospital, Emmen, the Netherlands); Anja U.G. van Lent (Department of Gastroenterology and Hepatology, Onze Lieve Vrouwe Hospital, Amsterdam, the Netherlands); Maartje Los (Department of Medical Oncology, St. Antonius Hospital, Nieuwegein, the Netherlands); Martijn R. Meijerink (Department of Radiology & Nuclear Medicine, Amsterdam University Medical Center—loc. VU, University of Amsterdam, Amsterdam, the Netherlands); Leonie J.M. Mekenkamp (Department of Medical Oncology, Medisch Spectrum Twente, Enschede, the Netherlands); Peter Nieboer (Department of Medical Oncology, Wilhelmina Hospital, Assen, the Netherlands); Koen C.M.J. Peeters (Department of Surgery, Leiden University Medical Center, University of Leiden, Leiden, the Netherlands); Natascha A.J.B. Peters (Department of Medical Oncology, Sint Jans Hospital, Weert, the Netherlands); Marco B. Polée (Department of Medical Oncology, Medical Center Leeuwarden, Leeuwarden, the Netherlands); Johannes F.M. Pruijt (Department of Medical Oncology, Jeroen Bosch Hospital, Den Bosch, the Netherlands); Patricia Quarles van Ufford-Mannesse (Department of Medical Oncology, Haga Hospital, Den Haag, the Netherlands); Ron C. Rietbroek (Department of Medical Oncology, Rode Kruis Hospital, Beverwijk, the Netherlands); Anandi H.W. Schiphorst (Department of Surgery, Diakonessenhuis Hospital, Utrecht, the Netherlands); Arjan Schouten van der Velden (Department of Surgery, St Jansdal Hospital, Harderwijk, the Netherlands); Ruud W.M. Schrauwen (Department of Gastroenterology and Hepatology, Bernhoven Hospital, Uden, the Netherlands); Mark P.S. Sie (Department of Medical Oncology, ZorgSaam Hospital, Terneuzen, the Netherlands); Lieke Simkens (Department of Medical Oncology, Maxima Medical Center, Eindhoven, the Netherlands); Dirkje W. Sommeijer (Department of Medical Oncology, Flevo Hospital, Almere, the Netherlands, and Department of Medical Oncology, Amsterdam University Medical Center—loc. AMC, University of Amsterdam, Amsterdam, the Netherlands); Dirk J.A. Sonneveld (Department of Surgery, Dijklander Hospital, Purmerend, the Netherlands); Leontine E.A. Spierings (Department of Medical Oncology, Alrijne Hospital, Leiderdorp, the Netherlands); Hein B.A.C. Stockmann (Department of Surgery, Spaarne Hospital, Haarlem, the Netherlands); Koen Talsma (Department of Surgery, Deventer Hospital, Deventer, the Netherlands); Frederiek Terheggen (Department of Medical Oncology, Bravis Hospital, Roosendaal, the Netherlands); Albert J. ten Tije (Department of Medical Oncology, Amphia Hospital, Breda, the Netherlands); Manuel L.R. Tjin-A-Ton (Department of Medical Oncology, Rivierenland Hospital, Tiel, the Netherlands); Renzo P. Veenstra (Department of Gastroenterology and Hepatology, Martini Hospital, Groningen, the Netherlands); Ankie M.T. van der Velden (Department of Medical Oncology, Tergooi Hospital, Hilversum, the Netherlands); Maarten Vermaas (Department of Surgery, Ijsselland Hospital, Capelle aan den Ijssel, the Netherlands); Wouter J. Vles (Department of Surgery, Ikazia Hospital, Rotterdam, the Netherlands); F. Jeroen Vogelaar (Department of Surgery, Viecuri Hospital, Venlo, the Netherlands); Theo van Voorthuizen (Department of Medical Oncology, Rijnstate Hospital, Arnhem, the Netherlands); Aad I. de Vos (Department of Medical Oncology, Van Weel-Bethesda Hospital, Dirksland, the Netherlands); Judith de Vos-Geelen (Department of Medical Oncology, Maastricht University Medical Center, Maastricht, the Netherlands); Johannes A. Wegdam (Department of Surgery, Elkerliek Hospital, Helmond, the Netherlands); Johannes H.W. de Wilt (Department of Surgery, Radboud University Medical Center, University of Nijmegen, Nijmegen, the Netherlands); David D.E. Zimmerman (Department of Surgery, Elisabeth-TweeSteden Hospital, Tilburg, the Netherlands).

The Prospective Dutch Colorectal Cancer (PLCRC) cohort is an initiative of the Dutch Colorectal Cancer Group (DCCG). PLCRC is supported by the Dutch Cancer Society; Stand Up to Cancer; ZonMw; Health Holland; Maag Lever Darm Stichting; Lilly (unrestricted grant); Merck (unrestricted grant); Bristol-Myers Squibb (unrestricted grant); Bayer (unrestricted grant); and Servier (unrestricted grant).

We would like to thank all patients who participated in this study.

**Author contributions:** Jeroen W.G. Derksen: Conceptualization, Methodology, Data curation, Formal analysis, Visualization, Writing—original draft. Anne M. May: Conceptualization, Methodology, Supervision, Writing—review & editing. Lonneke V. van de Poll-Franse: Conceptualization, Writing—review & editing. Belle H. de Rooij: Data curation, Formal analysis, Writing—review & editing. Dorothee Hafkenscheid: Writing—review & editing. Helena M. Verkooijen: Funding acquisition, Writing—review & editing. Miriam Koopman: Funding acquisition, Writing—review & editing. Geraldine R. Vink: Funding acquisition, Conceptualization, Supervision, Writing—review & editing.

## Data Availability

The data underlying this article were provided by the Netherlands Comprehensive Cancer Organisation (IKNL) and the Dutch Colorectal Cancer Group (DCCG) under license. Data will be shared on request to the corresponding author with permission of IKNL and the scientific board of the Prospective Dutch Colorectal Cancer Cohort/DCCG.

## Supplementary Material

pkab047_Supplementary_Data
