# Supplementary material for: Colorectal Cancer Care and Patients’ Perceptions Before and During COVID-19: Implications for Subsequent SARS-CoV-2 Infection Waves
Source: JNCI Cancer Spectr. 2021 May 13;5(4):pkab047. doi: 10.1093/jncics/pkab047 (PMC8243357; doi:10.1093/jncics/pkab047)
Supplement: pkab047_Supplementary_Data [file pkab047_supplementary_data.pdf]

SUPPLEMENTARY MATERIALS

Supplementary Methods

HRQoL questionnaire (pre- and during COVID-19)

For this analysis, we used 8 HRQoL scales, including global health status (score composed of two items, both with a 7-point ordinal scale ranging from 'very poor' to 'excellent'), five functional scales (i.e. physical, role, emotional, cognitive, and social functioning: five, two, four, two, and two items, respectively), one multi-item symptom scale (fatigue: three items), and one single-item symptom scale (sleep disturbances). Only during COVID-19, an additional single-item scales was used to assess worries about future health (obtained from EORTC library).

All items could be answered on a 4-point ordinal scale ranging from 'not at all' to 'very much'. Linear transformation was used to standardize the raw scores to scores ranging from 0 to 100, where a higher score represents a higher ('better') level of functioning or a higher ('worse') level of symptoms.

HADS questionnaire (pre- and during COVID-19)

This questionnaire consists of 14 items (7 anxiety-related and 7 depression-related), all answered on a 4-point Likert scale. Total scores range from 0 to 21, where a higher score represents more anxiety or depression. To identify a clinically relevant level of anxiety or depression, a cut-off score of 8 was used.

De Jong Gierveld short scales for loneliness questionnaire (only during COVID-19)

This questionnaire was used to measure social and emotional loneliness. All six items use a 3-point ordinal scale including 'no' (score 0), 'more or less' (1), and 'yes' (1). Patients were classified as not lonely (sum score 0-1), moderately lonely (score 2-4), or very lonely (5-6).

Categorization of sociodemographic data

Educational level	high, intermediate, low
Marital status/partnership	yes, no
Living situation	living alone, with partner and/or children
Body mass index	<25, 25-<30, ≥30 kg/m^2
Current presence of metastases	yes, no
Current treatment	radiotherapy, surgery, chemotherapy, immunotherapy, targeted therapy, active surveillance, symptom management, other
Presence of comorbidities	0, 1, ≥2 conditions

Supplementary Table 1. Changes cancer care planning and contact with health care professionals in case of physical complaints, questions or concerns during COVID-19

	Total population of CRC patients	CRC patients in follow-up	CRC patients in treatment	Matched control population	p-value
N (%)	3,247	2,456	594	1,114	
Contact with general practitioner (GP) during COVID-19 [a]					
Less quickly	571 (17.6)	464 (18.9)	67 (11.3)	240 (37.9)	<0.001
Equally quickly	2.336 (71.9)	1,739 (70.8)	468 (78.8)	368 (58.1)	
More quickly	41 (1.3)	29 (1.2)	8 (1.3)	17 (2.7)	
I never contact my GP myself	262 (8.1)	206 (8.4)	43 (7.2)	7 (1.1)	
N/a, currently no need to contact a GP [c]	-	-	-	481 (-)	
Contact with medical specialist/nurse during COVID-19 [b]					
Less quickly	397 (12.2)	344 (14.0)	38 (6.4)	156 (31.3)	<0.001
Equally quickly	2.036 (62.7)	1,478 (60.2)	473 (79.6)	232 (46.5)	
More quickly	30 (0.9)	15 (0.6)	15 (2.5)	8 (1.6)	
I never contact the hospital myself	737 (22.7)	590 (24.0)	60 (10.1)	103 (20.6)	
N/a, currently no need to contact the hospital [c]	-	-	-	615 (-)	
Patient-reported changes in cancer care planning					
Hospital visits					
- postponed and/or cancelled	454 (14.0)	363 (14.8)	86 (14.5)	-	n/a
- changed to TC/VC	395 (12.2)	259 (10.5)	128 (21.5)	-	
Treatments					
- adjusted	34 (5.7)	-	34 (5.7)	-	
- postponed	48 (8.1)	-	48 (8.1)	-	
- cancelled	4 (0.7)	-	4 (0.7)	-	

[a] Missing: N=18 patients in follow-up, N=8 patients in treatment. [b] Missing: N=29 patients in follow-up, N=8 patients in treatment. [c] This answer option was only available for the norm population and therefore not included in the statistical test, but only presented as count. Percentages do not always add up to 100 because of rounding to whole numbers. CRC = Colorectal cancer; GP = General practitioner; COVID-19 = Coronavirus disease 2019; TC = Telephone consultations; VC = Video consultations.

Supplementary Table 2. Factors associated with changes in global quality of life, anxiety and depression from <1 year pre- to during the COVID-19 pandemic in patients with CRC (N=2,338)

	Global quality of life (0-100) change (95% CI)	Anxiety (0-21) change (95% CI)	Depression (0-21) change (95% CI)
Age (year)	-0.05 (-0.12, 0.02)	0.01 (0.00, 0.03) *	0.02 (0.01, 0.03) **
Sex			
Male	ref.	ref.	ref.
Female	1.0 (-0.3, 2.3)	0.1 (-0.1, 0.3)	0.2 (0.0, 0.4) *
Marital status			
No partner	ref.	ref.	ref.
Partner	0.9 (-2.3, 4.1)	0.5 (0.0, 1.0)	0.1 (-0.4, 0.6)
Living situation			
Alone	ref.	ref.	ref.
With partner, no children	-0.3 (-3.3, 2.8)	-0.6 (-1.1, -0.1) *	-0.1 (-0.6, 0.4)
With partner and children	-0.2 (-4.0, 3.5)	-0.4 (-1.1, 0.2)	-0.1 (-0.7, 0.6)
No partner with children	-2.2 (-6.0, 1.5)	-0.4 (-1.0, 0.2)	0.1 (-0.5, 0.7)
Comorbidities			
0	ref.	ref.	ref.
1	0.4 (-1.2, 1.9)	0.2 (-0.1, 0.4)	0.1 (-0.2, 0.3)
2 or more	-1.0 (-2.5, 0.5)	0.0 (-0.3, 0.2)	0.0 (-0.2, 0.3)
Metastatic disease			
No	ref.	ref.	ref.
Yes	-0.2 (-2.2, 1.8)	-0.3 (-0.6, 0.1)	-0.4 (-0.8, -0.1) **
Treatment phase			
Completed treatment (follow-up)	ref.	ref.	ref.
Prior to or currently in treatment	-3.6 (-5.8, -1.4) **	0.0 (-0.3, 0.4)	0.6 (0.3, 1.0) **
Changes in cancer care			
No changes	ref.	ref.	ref.
Hospital visits cancelled, postponed or changed into TC/VC	0.3 (-1.3, 2.0)	0.1 (-0.1, 0.4)	-0.177 (-0.446, 0.093)
Treatment adjusted, postponed, or cancelled	1.5 (-1.3, 4.3)	0.3 (-0.2, 0.7)	0.075 (-0.384, 0.535)
Current supportive care			
No	ref.	ref.	ref.
Yes	0.4 (-1.0, 1.8)	0.1 (-0.2, 0.3)	0.2 (0.0, 0.5)
Worried about SARS-CoV-2 infection			
Not at all/a little	ref.	ref.	ref.
Quite a bit/very much	-3.1 (-4.6, -1.5) **	1.0 (0.7, 1.3) **	0.7 (0.5, 0.9) **

* p<0.05, ** p<0.01. CRC = Colorectal cancer; COVID-19 = Coronavirus disease 2019; TC = Telephone consultations; VC = Video consultations; SARS-CoV-2 = Severe Acute Respiratory Syndrome coronavirus 2.
